# Fast food proximity and weight gain in childhood and adolescence: Evidence from Great Britain

**DOI:** 10.1002/hec.4770

**Published:** 2023-11-16

**Authors:** Nicolás Libuy, David Church, George Ploubidis, Emla Fitzsimons

**Affiliations:** ^1^ Centre for Longitudinal Studies, Social Research Institute, UCL London UK

**Keywords:** health, obesity, population health

## Abstract

We study the relationship between proximity to fast food restaurants and weight gain from late childhood to early adolescence. We use the Millennium Cohort Study, a UK‐wide nationally representative longitudinal study, linked with granular geocoded food outlet data to measure the presence of fast food outlets around children's homes and schools from ages 7 to 14. We find that proximity to fast food outlets is associated with increased weight (body mass index, overweight, obese, body fat, weight), but only among those with maternal education below degree level. Within this sample, those with lower levels of emotional regulation are at heightened risk of weight gain.

## 1 INTRODUCTION

Recent decades have seen a surge in childhood obesity. In the UK, younger generations are at much higher risk of obesity, with the probability of being overweight 2–3 times higher for children born after the 1980s than before (Johnson et al., 2015). Similar trends have been observed in the US since the 1980s (Flegal & Troiano, 2000). Increasingly, the burden falls disproportionately on those from low‐income backgrounds (White et al., 2016).

Alongside this, the fast food retail industry in the UK, and globally, has seen a major expansion in recent decades. Fast food restaurants—including chip shops, burger bars, pizzerias—account for more than one quarter (26%) of all eateries in England (BBC, 2018; Cummins et al., 2005; Fraser et al., 2012b).^1^ Socioeconomic inequalities in takeaway food outlet density have also increased over time (Maguire et al., 2015), alongside inequalities in childhood obesity (Bann et al., 2018; NCMP, 2022).

A key question is the extent to which increased fast food supply is driving trends and inequalities in childhood weight. The majority of the evidence on this question comes from cross‐sectional data (Cobb et al., 2015; Feng et al., 2010; Fraser et al., 2012a; Harrison et al., 2011; Maguire et al., 2015; Mason et al., 2018; Snowdon, 2018; Walker et al., 2010; Williams et al., 2013), with the ensuing issue that associations may reflect socioeconomic deprivation, for instance, rather than the presence of fast food restaurants. A few papers attempt to estimate causal relationships, and are mostly confined to specific regions in the US (Alviola et al., 2014; Anderson & Matsa, 2011; Asirvatham et al., 2019; Currie et al., 2010; Davis & Carpenter, 2009; Dunn, 2010; Dunn et al., 2012; Powell, 2009; Qian et al., 2017), and Dolton and Tafesse (2022) in the UK. The general finding from the US literature is that the impact of fast food restaurants is significant but fairly small, with the one UK study finding no impact, suggesting fast food restaurants play a limited role in rising levels of childhood obesity. Most of the evidence examines proximity of fast foods to schools, rather than homes.

In this literature, there remains little evidence on the behavioral mechanisms that may be driving a relationship between fast food presence and childhood obesity. Changes in the food environment may induce changes in other health‐related behaviors. For instance, increased consumption of fast food may lead to compensatory increases in physical activity and/or reduced consumption of other less healthy foods. Understanding the mechanisms at play ‐ so which wider behavioral changes might be induced by policy change and how ‐ is important for informing and designing appropriate policy response. Identifying children most at risk is equally important for targeting, and a key outstanding question is whether certain groups are more susceptible to increased exposure to fast food outlets. There is some evidence that those from more disadvantaged backgrounds are at higher risk of obesity (Bann et al., 2018), but we know far less on the extent to which personal characteristics such as self‐regulation—a known correlate of drug abuse and binge drinking, and of eating disorders (Garland et al., 2018; Racine & Sarah, 2018; Weiss et al., 2015), and a determinant of life outcomes such as labor market success (Heckman et al., 2006; Kautz et al., 2014; Pearce et al., 2016)—play a role (Cawley, 2015). Emotional dysregulation in particular has been shown to predict excess weight gain and obesity among adolescents (Graziano et al., 2010; Kelly et al., 2016; Limbers & Summers, 2021). The lack of measures of both health behaviors and personal characteristics/skills has precluded exploration of this. One exception is Datar et al. (2023), who show that lower levels of patience play a role in increasing the likelihood of obesity.

In this paper, we use a rich longitudinal data set, the Millennium Cohort Study (MCS), linked with granular geographical data on the exact location of commercial and public facilities across Great Britain (the Ordnance Survey Point of interest [PoI] Dataset, hereon PoI), to examine how school and home proximity to fast food restaurants relates to children's weight (body mass index [BMI]), BMI standardized scores by sex, overweight and obesity, body fat and weight). We consider the period from mid‐childhood through early adolescence, which includes observations at ages 7, 11 and 14. The context is one where any purchase and consumption of snacks outside of school normally takes place on the journey to/from school, depending on local availability, with children gaining increased independence over this as they get older, and particularly on the transition from primary to secondary school at age 11.^2^ We also consider possible mechanisms, and in particular whether there are any behavioral changes in diet and exercise to compensate for increased exposure to fast foods. Finally, we explore heterogeneity in effects by sex and socioeconomic characteristics, along with the more novel domain of emotional regulation.

We use individual fixed effects (FE), exploiting changes in fast food restaurants near MCS respondents' homes and schools over time. We create more precise road network‐based buffers than in the extant literature (Bivoltsis et al., 2018) and examine three levels of proximity: 400, 800 and 1600 m, all within reasonable walking distance (Wilkins et al., 2019b). To reduce omitted variables bias, we use the rich longitudinal data to control for a range of time‐varying individual and area characteristics, and the PoI data to control for proximity to other food environments. We provide analyses of robustness of findings.

We find that increased proximity to fast food restaurants around children's homes results in increased BMI. Results are similar for other anthropometric measures including body fat, weight, BMI standardized scores by age, overweight and obesity. A one standard deviation increase in the number of fast food restaurants within 1600 m home‐buffer (or a total of 5.2 restaurants), that is, a 20‐min walking distance, increases BMI by 1.0% above the sample mean. The effect size decreases as proximity reduces—the further away from homes a fast food restaurant is located, the lower the effect on weight—consistent with increased transportation costs faced by consumers. A similar pattern emerges for children's schools—within the 1600 m buffer, a one standard deviation increase in fast food restaurants (or a total of 4.6 restaurants) increases BMI by 0.1 points, a 0.5% increase over the sample mean, and a similar effect of 0.6% is found for the 800 m buffer. However, for schools, we find that it is closer proximity—within 400–800 m—that is driving the detrimental effect on children's BMI.

While the effect on the overall sample is fairly low and consistent with what other studies have found, an interesting picture emerges when we examine heterogeneity. Compared with the overall effect, the effects of a one standard deviation increase in the 1600 m home‐buffer are almost twice as high among children with maternal education below degree level, at 1.7%. Around the 1600 m school‐buffer, it rises to 2% for children with maternal education below degree level, which is 3.7 times larger than the overall effect. We examine heterogeneity by levels of emotional regulation, using the emotional dysregulation sub‐scale of the Child Social Behaviors Questionnaire (Hogan et al., 1992; Sylva et al., 2004). For fast food exposure around the 1600 m home‐buffer, we find a greater increase in BMI for participants with lower emotional regulation. We have similar, though less precisely estimated, findings for the 1600 m school‐buffer.

We provide evidence to support our identification assumption that, conditional on individual FE and area‐ and individual‐level controls, the proximity of fast food restaurants to participants' homes and schools is as good as random. First, we show the presence of fast food restaurants is uncorrelated with time‐varying individual‐level characteristics, once we control for individual FE and area‐level variables. Second, we show that our results are robust to controlling for several individual time‐varying variables and area‐level time‐varying economic conditions that may affect trends in the fast food markets. We also show that the number of fast food restaurants in areas near where children live is not correlated with changes in residence, and that our results are not driven by changes in the supply of fast food restaurants among the subset of movers in our sample, providing some evidence that selection into geographical areas is not a major concern in our sample. Our results are also robust to controlling for mode of transport from home to school, as well as to changes in the classification of fast food restaurants. We address concerns about the validity of our two‐way FE identification strategy by replicating our main results using the estimator proposed by de Chaisemartin and D’Haultfœuille (2020), that allows for heterogeneity in treatment effects across time and treated units. Finally, we perform falsification tests and examine the relationship between children's weight and increases in the presence of other services such as in construction and employment agencies, which we would not expect to be correlated with outcomes.

We explore two mechanisms that may explain our findings of limited impacts, relating to diet and physical activity. In response to children's increased fast food consumption, parents may change other aspects of their children's diet to make it healthier, and/or may increase their physical activity. We study whether fast food exposure induces any compensating improvements in other aspects of children's dietary quality, relating to consumption of fruits, breakfast, and sweetened drinks, finding null effects. We also find no evidence that fast food proximity increases the take‐up of meals provided by schools, which meet quality nutrition standards. Regarding exercise, we do not find that an increase in the presence of fast food restaurants induces compensatory behaviors in children's physical activity.

In providing among the first evidence for Great Britain, our study makes an important contribution to a more US‐focused literature (Alviola et al., 2014; Asirvatham et al., 2019; Currie et al., 2010; Dunn, 2010; Qian et al., 2017; Zeng et al., 2019). It is also one of the first to investigate the extent to which the low estimated effect of fast food proximity on children's weight may reflect compensatory behaviors, and in particular improvements in other aspects of diet and exercise. In addition, our novel examination of heterogeneity, most notably as a function of emotional regulation, allows us to understand further the role of demand factors, in the same spirit as Allcott et al. (2019). The ability to regulate emotions has been shown to drive the demand for healthy behaviors, including drug intake and food consumption (Garland, 2018; Racine & Sarah, 2018; Weiss et al., 2015). We show that it is a moderator in determining the weight inequalities we observe, providing new insights into the ongoing discussion around geographical nutritional disparities in the “food deserts” literature (Allcott et al., 2019; Bitler & Haider, 2011). Finally, in examining proximity of fast food restaurants to both schools and homes separately, we compare our results on obesity and BMI with previous literature, which mainly focuses on proximity to schools. Studies that examine the impact of fast food restaurants near schools on childhood obesity find positive and relatively small effects (Alviola et al., 2014; Asirvatham et al., 2019; Currie et al., 2010; Davis & Carpenter, 2009; Zeng et al., 2019), which are similar to the positive effects found for fast food restaurants near homes (Qian et al., 2017), but we are not aware of any studies than examine both.

The paper is organized as follows. In Section 2, we describe the data and present summary statistics. Section 3 presents our methodology and identification strategy. We present our results in Section 4, robustness checks in Section 5 and conclude in Section 5.

## 2 DATA AND SUMMARY STATISTICS

We combine longitudinal data from the MCS with highly granular geographical data from the PoI and Ordnance Survey Integrated Transport Network (ITN) to create measures of the number of fast food and other retail food establishments within 400, 800, and 1600 m network buffer zones around each respondent's home and school postcodes.

### 2.1 Millennium Cohort Study

The MCS is a longitudinal study following the lives of a nationally representative sample of 19,244 families with children born between 2000 and early 2002 in the UK (Joshi & Fitzsimons, 2016). Starting when children were 9 months old, and then at ages 3, 5, 7, 11, 14 and 17,^3^ the MCS collects extensive information on respondents and their families, including parental education; employment and income; housing; family structure; ethnicity; physical and mental health, and health behaviors; cognitive and physical development, among many other behaviors and characteristics.

We examine BMI, and also present evidence for body fat and other anthropometric variables because together they provide a more accurate picture of adiposity (Nuttall, 2015).^4^ BMI is calculated by dividing weight in kilograms by squared height in meters. Height was measured using Leicester height stadiometers^5^ and recorded to the nearest millimeter. Weight and body fat measurements were taken by trained interviewers using Tanita BF‐522W scales, which calculate weight to the nearest 0.1 kg, and body fat percentage to the nearest 0.1%. The percentage of body fat was calculated measuring the amount of resistance encountered by a weak electrical current as it travels through the body (Chaplin Grey et al., 2010).

### 2.2 Ordnance survey PoI

The PoI data contain geocoded information on over 4 million commercial and public facilities across Great Britain, and is available annually from 2005 to 2013, and quarterly from September 2014 (OS, 2015). Each facility is geocoded and assigned to one of around 600 classes, which are further classified into larger categories and groups, resulting in an extremely rich and granular data set.^6^

PoI has a high level of correspondence with street level audits, regarded as the gold standard for spatial data (Wilkins et al., 2017). It is a validated dataset (Burgoine & Harrison, 2013), with high spatial and count accuracy, especially post‐2010,^7^ and has been used in UK Retail Food Environment (RFE) research (Burgoine et al., 2017; Cetateanu & Jones, 2014; Harrison et al., 2011; Jennings et al., 2011; Fraser et al., 2012a; Skidmore et al., 2010). We extract data on food outlets, following previous work, to characterize obesogenic environments (Cetateanu & Jones, 2014; Jennings et al., 2011; PHE, 2016, 2017; Skidmore et al., 2010). We describe this in detail in Section 2.4.

### 2.3 Ordnance survey ITN

The ITN dataset is a snapshot of the entire road network of Great Britain, containing detailed road‐routing information. ITN data are available from 1997 onwards, on an annual or biannual basis.^8^ We use the ITN dataset around each respondent's home and school postcodes at each sweep^9^ to construct road network‐based buffer, which account for how the road network affects individual travel. These highly local irregular buffers, which have been extensively used in the fast food literature, better characterize the respondent's neighborhood considering that individuals often cross administrative boundaries for food (Charreire et al., 2010). Moreover compared to Euclidean distance, road network‐based buffers are considered to provide a more accurate characterization of the influence of the built environment (Bivoltsis et al., 2018; Charreire et al., 2010). We use a Geographic Information System to construct 400, 800, and 1600 m road network‐based buffers, which are the most prevalent in RFE research (Wilkins et al., 2019b), since they equate to an average person's 5‐, 10‐, and 20‐min walking distance respectively.

### 2.4 Classification of fast food and other outlets

We use PoI data from Septembers of 2008, 2012 and 2015, and ITN data from October 2008, June 2012, and June 2015 in order to overlap with the age 7, 11 and 14 sweeps of the MCS.

To characterize obesogenic environments across time and locations we count the number of fast food restaurants and other food outlets within road network‐based buffers around respondents' schools and residences. In the absence of any standardized food classification schemas (Block et al., 2018), some research, all US‐based, has categorized fast‐food outlets based on the biggest/most popular national chains (e.g., Alviola et al., 2014; Currie et al., 2010; Davis & Carpenter, 2009; Dunn, 2010; Dunn et al., 2012; Qian et al., 2017; Zeng et al., 2019). UK work tends to use PoI categories such as “Fast food and takeaway outlets” and “Fast food and delivery services” (Cetateanu & Jones, 2014), but other classifications based only on popularity and geographic presence have also been used (Robinson et al., 2018). In addition, classifying food retailers according to their “healthfulness” is not straightforward (Pinho et al., 2019). For example, even though supermarkets are usually considered to be a source of healthy foods (Woodruff et al., 2018), they also offer a wide range of sugar‐saturated beverages and snack foods. Correspondingly, many major fast food restaurants also offer healthier choices (Mahendra et al., 2017).

For this study, relying only on the “Fast food and takeaway outlets” and “Fast food and delivery services” categories of the PoI data to evaluate changes over time is challenging because some major fast food chains were not appropriately included in these categories before 2010.^10^ To avoid inconsistencies in the classification of fast food restaurants between data collected before and after 2010, our classification of fast food restaurants combines both schemes used in the previous literate: biggest/most popular fast food chains and food categories in PoI data that were consistently recorded over time. We first include the major UK fast food restaurants (based on Robinson et al., 2018; Wilkins et al., 2019a): McDonalds, KFC, Burger King, Wimpy, Subway, Pizza Hut, and Dominos' Pizza.^11^ These are identified straightforwardly using the name recorded in the PoI data. We also include fish and chips shops, a traditional take‐away food in the UK, identified using the available category in the PoI data, and kebab and chicken outlets, identified through restaurants classified as food outlets and containing the words “Kebab” and/or “Chicken” in their name. We include more details of our classification in the Appendix.

We also measure the number of other food outlets within the buffers, using the other food facilities in the PoI data, as the presence of both healthy and unhealthy food has been found to be associated with dietary habits and BMI in cross‐sectional analyses (Burgoine et al., 2014; Hobbs et al., 2019; Fraser et al., 2012a). Moreover, other food facilities around children's schools and residences are a good proxy for neighborhood characteristics that are correlated with factors that both contribute to obesity and to the presence of fast food restaurants (Currie et al., 2010). Our classification of other food facilities includes: restaurants, butchers, confectioners, delicatessens, fishmongers, green and new age goods, grocers, farm shops and pick your own, organic and health foods, gourmet and kosher foods, convenience stores and independent supermarkets, and supermarket chains, other take away outlets.

## 3 METHODOLOGY

We estimate FE models to study the relationship between fast food restaurant proximity and BMI. We provide results for additional anthropometric variables—body fat, weight, BMI standardized scores by age, overweight and obese—in Table A4. We estimate two models:

(1) $$Y_{it}=\beta^{k}F_{it}^{k}+X_{it}^{´}\rho^{k}+Z_{at}^{´}\delta^{k}+\alpha_{i}^{k}+\eta_{t}^{k}+\varepsilon_{it}^{k}$$

where *Y*_*it*_ is the BMI of individual *i* at ages *t* = 7, 11 and 14. In model (1), $F_{it}^{k}$ is the number of fast food restaurants within distance *k* of respondent i's location at age *t*. We estimate the models separately for home and school locations, and we run three separate specifications for each—for *k* = 400, 800, and 1600 m. Large buffers overlap small ones. $\alpha_{i}^{k}$ is an individual fixed effect and $\eta_{t}^{k}$ is a year of survey fixed effect. $X_{it}^{´}$ includes a range of socioeconomic characteristics of families, including six dummies indicating the maternal highest educational level at time of interview,^12^ number of parents in the household, family income at time of interview, total household size, number of rooms in the household, household tenure, and individual's age and age squared. Age varies within a sweep depending on date of interview. $Z_{at}^{´}$ includes the local authority district^13^ unemployment rate and population estimates per 100,000 people to control for time‐varying economic conditions at the area (a) level that may affect the fast food industry and also be associated childhood weight. $\varepsilon_{it}^{k}$ is a disturbance error assumed to be independent and identically distributed. Our parameter of interest in Equation (1) is *β* ^*k*^.

To study how the effect varies as the buffer changes, we estimate

(2) $$Y_{it}=\gamma_{1}F_{it}^{400}+\gamma_{2}F_{it}^{800}+\gamma_{3}F_{it}^{1600}+X_{it}^{´}\rho +Z_{at}^{´}\delta +\alpha_{i}+\eta_{t}+\varepsilon_{it}$$

In Equation (2), $F_{it}^{400}$, $F_{it}^{800}$, and $F_{it}^{1600}$ are non‐overlapping buffers denoting the number of fast food restaurants within a 400 m buffer of the individual i's location at age *t*, between 400 and 800 m, and between 800 and 1600 m, respectively. Our key parameters of interest are *γ*_1_, *γ*_2_, and *γ*_3_.

To account for food outlets other than fast foods affecting weight, the $X_{it}^{´}$ vector also includes the number of other food outlets as described previously.^14^ In addition, the inclusion of other food outlets around children's residences and schools helps mitigate the influence of local neighborhood characteristics that are unobserved but that may be correlated both with the presence of fast food and with unobserved factors that contribute to childhood obesity. We cluster standard errors at the individual level, and estimates are weighted to account for attrition, using inverse probability weights, and survey design (Solon et al., 2015).

The effect of fast food presence on children's BMI is identified by changes in the number of fast food restaurants over time. The identification assumption is that, conditional on individual FE, year of survey dummies, and time‐varying controls, no time‐varying unobserved variables are systematically correlated both with changes in BMI and changes in the number of fast food restaurants, which we probe more extensively in the latter part of Section 5.

## 4 RESULTS

We first provide summary statistics pertaining to the sample, then present our main results and an analysis of the plausibility of our identification strategy. We also explore heterogeneity in effects, showing estimates for different subsamples, and discuss potential mechanisms that may be at play. Finally, we present a series of robustness checks and falsification tests.

### 4.1 Summary statistics

We base our analysis on MCS respondents interviewed at ages 7, 11 and 14, including only those with valid measurements of BMI and body fat percentage through this period (92% of MCS respondents). We exclude Northern Ireland, given the absence of PoI data. Our analytical sample includes 8253 children. Table 1 presents the mean and standard deviation of child excess weight across time in the sample.^15^ Around 18% of 7‐year‐olds were overweight and a further 4.7% obese, with an average BMI of 16.4, and body fat percentage 20.7%. Four years later, the percentage of children overweight and the percent of body fat increased to 25.7% and 22.0%, respectively, remaining stable to age 14 despite a small increase in BMI from 19.1 to 21.4.

**TABLE 1:** Descriptive statistics: Anthropometric measurements and fast food supply.

	7 years		11 years		14 years		Full sample	
	Mean or %	Std. dev.	Mean or %	Std. dev.	Mean or %	Std. dev.	Mean or %	Std. dev.
Weight in kilograms	25.2	4.7	41.1	9.7	58.0	13.0	41.4	16.6
Height in centimeters	123.5	5.5	146.2	7.2	164.2	8.1	144.6	18.1
Body Mass Index	16.4	2.2	19.1	3.5	21.4	4.1	19.0	4.0
Percentage of body fat	20.7	5.2	22.0	7.8	21.7	9.2	21.5	7.6
Child is obese	4.7		5.8		7.3		5.9	
Child is overweight	18.0		25.7		26.1		23.3	
Fast food restaurants around homes								
1600 m	2.5	3.3	4.1	6.0	4.0	5.7	3.5	5.2
800 m	0.6	1.1	1.0	2.0	1.0	1.9	0.9	1.7
400 m	0.2	0.5	0.2	0.7	0.2	0.7	0.2	0.6
Fast food restaurants around schools								
1600 m	2.7	3.3	3.9	5.4	3.8	4.9	3.5	4.6
800 m	0.7	1.2	1.1	1.9	0.8	1.5	0.9	1.6
400 m	0.2	0.5	0.2	0.6	0.1	0.5	0.2	0.5
Observations	8253		8253		8253		24,759	

*Note*: The overweight and obese categories are defined using the International Obesity Task Force (IOTF) cutoffs (Cole et al., 2000).

Tables A1 and A2 present the average number of fast food restaurants and other food outlets around children's residences and schools over time. Several interesting facts emerge. First, we observe an increase over time in the number of fast food restaurants around children's homes and schools: at age 7, children had an average of 2.5 fast food restaurants within 1600 m of home, while 7 years later the number had increased by around 60%, to 4. This trend is also observed in relation to children's schools, where fast food restaurants within 1600 m increased by over 40% during this period, from 2.7 to 3.8. Second, not only is the number of fast food restaurants increasing during this period, but so too are other food facilities. On average, at age 7 there were around 20.1 (21.5) other food facilities within 1600 m of children's homes (schools). By age 14, these numbers had increased by 46.3% (40.9%).

Table 2 shows descriptive statistics for the overall analytical sample (column 1), and by distance from home (or school) to fast food restaurants. Around 67.8% (71.8%) of children have lived (attended school) within 1600 m, or 1 mile, of fast food restaurants during the analytic period.

**TABLE 2:** Descriptive statistics by presence of fast food restaurants (%).

		Home			School		
	All	<1600 mts.	<800 mts.	<400 mts.	<1600 mts.	<800 mts.	<400 mts.
	(1)	(2)	(3)	(4)	(5)	(6)	(7)
Demographics							
Child is male	52.0	51.8	51.7	53.4	51.8	51.9	52.0
Mother is white ethnicity^b^	84.4	79.7	75.4	72.9	80.5	78.6	78.8
Mother is mixed ethnicity	3.3	3.9	3.8	3.6	3.7	3.3	3.9
Mother is Indian ethnicity	2.3	2.8	3.4	3.1	2.8	2.8	2.9
Mother is Pakistani or Bangladeshi ethnicity	4.6	6.1	8.6	11.5	6.0	7.0	7.8
Mother is black or black British ethnicity	4.0	5.4	6.8	6.7	5.2	6.2	4.9
Mother is from another ethnic group	1.4	2.0	1.9	2.3	1.9	2.1	1.7
Mother highest NVQ level is 1^a^ ^,^ ^b^	8.1	8.7	9.4	10.9	8.7	9.5	11.9
Mother highest NVQ level is 2^a^	28.4	28.5	29.0	27.3	29.0	27.1	27.1
Mother highest NVQ level is 3^a^	14.7	14.4	13.5	13.0	14.6	14.9	15.2
Mother highest NVQ level is 4^a^	28.1	25.5	23.4	23.4	25.3	24.0	24.2
Mother highest NVQ level is 5^a^	5.2	5.2	5.1	4.8	5.2	4.9	4.5
Mother has overseas qualification only^a^	3.2	3.5	3.5	3.4	3.6	4.1	3.3
Mother does not have any of these qualification^a^	12.1	14.3	16.0	17.2	13.7	15.5	13.7
Number of parents/carers in household							
Two parents/carers^a^ ^,^ ^b^	77.9	76.1	74.7	73.0	76.3	75.3	76.1
One parent/carer^a^	22.1	23.9	25.3	27.0	23.7	24.7	23.9
OECD equivalised weekly family income^a^	387.4	365.0	342.2	319.6	370.2	357.4	355.1
Number of people in household (not including individual)^a^	3.5	3.5	3.6	3.6	3.5	3.6	3.6
Numbers of rooms in the household^a^	6.0	5.8	5.7	5.6	5.9	5.8	5.9
Housing tenure							
Own‐mortgage/loan^a^ ^,^ ^b^	55.6	52.8	50.2	45.9	53.8	51.8	53.7
Own outright^a^	5.5	5.1	5.1	5.1	5.1	5.2	5.8
Rent or other^a^	38.9	42.1	44.7	49.0	41.0	43.0	40.5
Local authority level variables							
Unemployment rate^a^	8.0	8.4	8.7	8.8	8.3	8.5	8.6
Population estimates^a^	2.3	2.5	2.6	2.7	2.4	2.4	2.4
Observations	8253	5603	3054	1060	5929	3624	1385

*Note*: This table shows descriptive statistics at age 7 by the presence of fast food restaurants within 400, 800, 1600 m buffers. Columns 2 & 5; 3 & 6; and 4 & 7 include children with one or more fast food restaurants within 1600, 800, 400 m buffers, respectively.

^a^ Indicates time‐varying variables included as controls in Equations (1) and (2).

^b^ Indicates the baseline category excluded in the empirical specifications. Descriptive statistics of the variables *Fast food restaurants* and *Other food outlets* are in the Appendix.

### 4.2 The impact of fast food restaurants on children's BMI

We first estimate Equations (1) and (2) separately for children's homes and schools. Table 3 presents our preferred within‐individual estimates that capture whether changes in children's BMI are affected by changes in the number of fast food restaurants around children's homes and schools. We show cross‐sectional estimates for BMI and other anthropometric measures in Table A3. Columns 1 and 5 show the results around homes and schools for a specification that includes only year of survey and individual FE. Overall, the results show that the impact of an additional fast food restaurant within 1600 m of respondents' homes on BMI is positive and significant. We also find an increased number of fast food restaurants around children's schools increases BMI, mainly driven by the 800 m buffer. The positive association between the number of fast food restaurants around homes and schools and BMI is robust to the inclusion of the number of other food outlets (columns 2, 6), time‐varying individual controls (columns 3, 7), and local authority district controls (columns 4, 8). We find similar results when we use as outcomes the percentage of body fat, weight in kilograms and BMI standardized scores (Table A4).

**TABLE 3:** The impact of fast food restaurants on BMI.

	Home				School			
	(1)	(2)	(3)	(4)	(5)	(6)	(7)	(8)
Panel A. Fast food restaurant within								
Equation (1), *k* = 400 m	0.1140*	0.1030	0.1000	0.0980	−0.0061	0.0341	0.0318	0.0296
	(0.0594)	(0.0794)	(0.0777)	(0.0775)	(0.0342)	(0.0432)	(0.0428)	(0.0426)
Equation (1), *k* = 800 m	0.0574***	0.0607**	0.0591**	0.0554**	0.0291**	0.0750***	0.0741***	0.0742***
	(0.0200)	(0.0257)	(0.0255)	(0.0255)	(0.0143)	(0.0236)	(0.0229)	(0.0229)
Equation (1), *k* = 1600 m	0.0290***	0.0389***	0.0390***	0.0355***	0.0022	0.0220**	0.0218**	0.0225**
	(0.0088)	(0.0127)	(0.0126)	(0.0126)	(0.0088)	(0.0107)	(0.0106)	(0.0106)
Panel B. Equation (2)								
Fast food restaurant								
Within 400 m	0.0921	0.108	0.1040	0.1020	−0.0165	0.0446	0.0424	0.0404
	(0.0601)	(0.0791)	(0.0774)	(0.0773)	(0.0342)	(0.0432)	(0.0428)	(0.0426)
Between 400 and 800 m	0.0292	0.0476	0.0472	0.0432	0.0444***	0.0819***	0.0813***	0.0818***
	(0.0272)	(0.0309)	(0.0306)	(0.0306)	(0.0158)	(0.0251)	(0.0244)	(0.0244)
Between 800 and 1600 m	0.0243**	0.0312**	0.0317**	0.0283**	−0.0083	0.0059	0.0059	0.0068
	(0.0110)	(0.0141)	(0.0142)	(0.0142)	(0.0111)	(0.0121)	(0.0121)	(0.0120)
Other food outlets	No	Yes	Yes	Yes	No	Yes	Yes	Yes
Individual controls	No	No	Yes	Yes	No	No	Yes	Yes
Area‐level controls	No	No	No	Yes	No	No	No	Yes
Individual FE	Yes	Yes	Yes	Yes	Yes	Yes	Yes	Yes
Year of survey FE	Yes	Yes	Yes	Yes	Yes	Yes	Yes	Yes
Observations	24,759	24,759	24,759	24,759	24,759	24,759	24,759	24,759
Number of children	8253	8253	8253	8253	8253	8253	8253	8253
Mean of dependent variable	18.95	18.95	18.95	18.95	18.95	18.95	18.95	18.95

*Note*: Columns 1–4 show estimates around respondents' residences and columns 5–8 around schools. In Panel A, each cell reports a different regression, and rows show results for three different equations—one for each respective buffer indicated in Equation (1): *β* ^400^, *β* ^800^, and *β* ^1600^. In Panel B, rows show estimates of Equation (2): *γ*_1_, *γ*_2_, and *γ*_3_. Other estimates in Equations (1) and (2) are omitted due to space limitations but available upon request. ***, **, and * denote statistically significant at 1%, 5% and 10%. Robust standard errors in parenthesis are clustered at the individual level.

Looking more closely at the estimates, and first at those pertaining to proximity of fast foods to homes (columns 1–4 in Panel A of Table 3), an additional fast food restaurant within 1600 m, from ages 7 to 14, increases respondents' BMI by 0.036 points, or a 0.2% increase over the sample mean of 18.95. The estimates are a little larger when we focus on fast food restaurants within 800 m, with an increase of 0.3% with respect the sample mean. The size of the estimates decreases as the buffer radius increases, consistent with higher transportation and psychological costs faced by individuals (Currie et al., 2010). Results around children's schools (columns 5–8 in Panel A of Table 3) show a similar pattern but are slightly larger, with an additional fast food restaurant within 1600 (800) meters increasing BMI by 0.5% (0.6%) over the sample mean.

Estimates from Equation (2) in Panel B show that the impact around homes is likely driven by the increase in fast food restaurants between 800 and 1600 m (column 4). Although point estimates within 400 m and between 400 and 800 m are relatively larger than 800–1600 m, they are less precisely estimated. Interestingly, the findings around children's schools are mainly driven by increases in fast food restaurants between 400 and 800 m (column 8). This evidence suggests that close proximity of fast food restaurants to schools is more detrimental than to homes.

In terms of the magnitude of the associations, our main specification that shows an additional fast food restaurant—respectively—within 400, 800, and 1600 m of children's homes increases BMI by 0.10, 0.06, 0.04 points, translates into a gain of 344, 157, and 86 g during the period under consideration. Expressed in standardized BMI scores, these estimates are 0.029, 0.018, and 0.008, respectively (see column 3 in Table A4), with the last two statistically significant at the 5% and 10% level. To compare to existing estimates, Qian et al. (2017), find that the effect of an additional fast food restaurant within 1600 m of the child's residence on BMI standardized scores was 0.0019, whilst Asirvatham et al. (2019) find null effects. Previous studies, using school‐level data in Arkansas and instrumental variables, find that an additional fast food restaurant within 1600 m increases the obesity rate by 1.23% (Alviola et al., 2014) and 1.22% (Qian et al., 2017). By comparison, in the same 1600 m buffer around schools, we find an increase of 0.2 percentage points in obesity, a 3.4% increase with respect to a sample rate of 5.8%.^16^

### 4.3 Transmission mechanisms

Our overall finding is that fast food presence has a limited impact on children's weight, and one potential explanation for this is that we may be estimating an effect that also reflects other behavioral responses to fast food proximity: children may improve other aspects of their diets and/or increase their levels of physical activity, as a possible (parental‐led) response to increased access to fast foods. Understanding the extent to which this may be at play is important for formulating appropriate policy response.

We examine two dimensions of diet, as proxies for less healthy diets—consumption of particular foods (low fruit, regular sweetened drinks, skipping breakfast) and take‐up of school meals, which meet national nutritional standards.^17^ We also create a score variable to proxy *unhealthy diet habits*, the sum of the first three binary variables, with higher scores indicating lower quality diets.

Estimates in Table A13 provide no evidence that an increased presence of fast food restaurants around children's homes leads to improvements in other aspects of an individual's diet, by way of compensation.^18^ Whilst this is informative, these results should be interpreted carefully owing to limitations in our measures, including that they only measure the extensive margin, and not more frequent changes in behaviors, providing only a partial characterization of dietary behaviors.

We next study whether proximity to fast food restaurants around homes and schools affects whether or not participants receive school meals. Access to free school meals is determined by parental eligibility for certain benefits, and non‐eligible families can choose to avail of school meals by paying for them. Around 16% of children in our sample at age 7 received free school meals, and a further 39% paid for school meals. Given that school meals in the UK have to meet high quality nutritional standards (Evans & Harper, 2009), our hypothesis is that the presence of unhealthy food options such as fast food restaurants could induce parental compensatory behaviors to increase the take‐up of school meals. The null results in column 5 of Table A13 indicate that this is not at play, however.

We next examine whether physical activity changes in the presence of fast food restaurants: if higher fast food presence induces an increase in the consumption of fast food, then individuals may respond by increasing their levels of physical activity—mitigating the overall impact on their BMI. We estimate Equation (1) using as an outcome the weekly frequency of physical activity. We define three binary variables that indicate whether the individual exercised 1 or more, 3 or more, and 5 or more days per week. We also create a continuous variable that imputes the mid‐point of the intervals associated with each category (i.e., 0, 1.5, 3.5, and 6 days). Findings in Table A14 provide no evidence that changes in food environments induce children to change their physical activity.

### 4.4 Heterogeneity

We now examine whether the effect of fast food restaurants on BMI varies by sex and socioeconomic status, as well as by levels of emotional regulation. The latter has been shown to be associated with a host of risky behaviors and obesity among adolescents (Graziano et al., 2010; Kelly et al., 2016; Limbers & Summers, 2021), along with major domains of life including in employment and education (Kautz, 2014). To test this, we estimate Equation (1) and interact our fast food restaurant and other food outlet variables with these characteristics. Columns 1–4 (5–7) of Table 4 show estimates for the 1600 m buffer around children's homes (schools).

**TABLE 4:** Heterogeneity in the effect of fast food restaurants on BMI.

	Home				School			
	(1)	(2)	(3)	(4)	(5)	(6)	(7)	(8)
Fast food restaurant								
Within 1600 m	0.0325*	−0.00844	0.0142	−0.0726	0.0433***	−0.0249**	0.0161	−0.0131
	(0.0178)	(0.0157)	(0.0205)	(0.0505)	(0.0162)	(0.0108)	(0.0126)	(0.0416)
Within 1600 m × male	0.00233				−0.0403*			
	(0.0244)				(0.0212)			
Within 1600 m × low education		0.0618***				0.0841***		
		(0.0228)				(0.0196)		
Within 1600 m × high emotional dysregulation			0.0488*				0.0185	
			(0.0265)				(0.0219)	
Within 1600 m × emotional dysregulation score				0.0640**				0.0222
				(0.0275)				(0.0259)
Other food outlets	Yes	Yes	Yes	Yes	Yes	Yes	Yes	Yes
Individual controls	Yes	Yes	Yes	Yes	Yes	Yes	Yes	Yes
Area‐level controls	Yes	Yes	Yes	Yes	Yes	Yes	Yes	Yes
Individual FE	Yes	Yes	Yes	Yes	Yes	Yes	Yes	Yes
Year of survey FE	Yes	Yes	Yes	Yes	Yes	Yes	Yes	Yes
Observations	24,759	24,759	24,258	24,258	24,759	24,759	24,258	24,258
Number of children	8253	8253	8086	8086	8253	8253	8086	8086
Mean of dependent variable	18.95	18.95	18.95	18.95	18.95	18.95	18.95	18.95

*Note*: This table shows OLS results for a modified Equation (1) that interacts the fast food restaurants and other food outlets variables with the variables shown in the rows. Male is a dummy variable that takes value 1 if child is male and 0 if is female. Low educational level is a dummy variable equal to 1 if the highest maternal education (measured when the child was 5 years old) is below degree level and 0 if it is degree level or higher. High emotional dysregulation is a dummy variable equal to 1 if the emotional dysregulation sub‐scale of the Child Social Behaviors Questionnaire at age 7 is above the sample median and 0 otherwise. The Emotional dysregulation score is the score of the dysregulation sub‐scale of the Child Social Behaviors Questionnaire at age 7. Each column reports a different regression. Other estimates in Equation (1) are omitted due to space limitations but are available upon request. ***, **, and * denote statistically significant at 1%, 5% and 10%. Robust standard errors in parenthesis are clustered at the individual level.

#### 4.4.1 Sex

Previous research has documented sex‐specific attitudes toward dietary behaviors, and differences in risk attitudes and behaviors have been observed between males and females (Eckel & Grossman, 2002). We find no differences in effects between males and females for fast food restaurants around homes (columns 1 in Table 4), and smaller (although less precisely estimated) effects for males than females around schools.

#### 4.4.2 Socioeconomic status

Previous research portrays a clear pattern of disparity in adolescent obesity by socioeconomic status (Bann et al., 2018). An important question, not addressed in previous studies, is the extent to which the presence of fast food restaurants might exacerbate these inequalities. We examine heterogeneity by maternal education level—specifically, whether the individual's mother has a degree or higher (47.7%) or not. The impact of fast food restaurants on BMI is significant and almost twice as high among children whose mothers have lower education levels (columns 2 and 4 in Table 4), indicating that fast food exposure could exacerbate nutritional inequalities.

#### 4.4.3 Self‐regulation

We explore whether a lower ability to regulate emotional responses, that is, emotional dysregulation, may play a role in determining the impact of fast food restaurants on BMI. Emotional dysregulation involves, among other things, a lower capability to control impulsive behaviors and has been associated with adolescent obesity (Kelly et al., 2016; Limbers & Summers, 2021) and a range of risky behaviors such as substance use (Garland, 2018; Weiss et al., 2015), and mental illness including self‐harm (Crowell, 2018) and eating disorders (Racine & Sarah, 2018). Additionally, eating behaviors such as emotional eating, that is, overeating in response to negative emotions, has been considered a marker of emotional dysregulation, and one that could help in clinically screening early obesity diagnoses (Micanti et al., 2017).

We use the emotional dysregulation sub‐scale of the Child Social Behaviors Questionnaire at age 7 (Hogan et al., 1992; Sylva et al., 2004). We first validate our measure of emotional dysregulation by analyzing whether high levels of emotional dysregulation at age 7 predict fast food consumption at age 14, risk behaviors (smoking and drinking) at 14, patience at 14, and risk taking at 11. Fast food consumption is a binary variable that measures whether the individual eats fast food one or more days per week. Smoking and drinking are binary variable indicating if the individual has ever smoked or drunk alcohol. We use the question “how patient would you say you are?” to measure patience at age 14, a score ranging from 0 to 10, where 0 is never and 10 is always. Risk taking at age 11 is measured using the risk taking score of the CANTAB Cambridge Gambling Task, where higher values indicate of greater risk taking (Atkinson, 2015). Table A12 shows Ordinary Least Squares estimates for outcomes at age 11 and 14 as a function of respondents' emotional dysregulation at age 7, controlling for individual and local area variables at age 7. Results reveal a consistent pattern, whereby children with high emotional dysregulation at age 7 are more likely to eat fast food, more likely to have ever tried smoking, ever tried alcohol, more impatient, and more willing to take risk.

We next analyze whether the effect of fast food restaurants presence on BMI varies by emotional dysregulation. Results are shown in Table 4. The effect is larger for those with higher levels of emotional dysregulation, suggesting that proximity to fast foods is more detrimental for those with a lower ability to self‐regulate. When we study the effects by low/high maternal education and low/high emotional regulation, we find that the impact of a one standard deviation (or 5.2 restaurants) increase in fast food exposure on BMI is 0.54 points (95% CI: 0.24, 0.83) larger for more at‐risk participants (low maternal education and low emotional regulation) than less vulnerable participants (high maternal education and high emotional regulation).^19^

## 5 ROBUSTNESS

The impact of fast food restaurant proximity on children's BMI is identified under the assumption that, conditional on individual FE and area‐ and individual‐level controls, the presence of fast food restaurants near MCS participants' residences and schools is as good as random. Estimates could be biased if the openings and closures of fast food restaurants respond to changes in families' preferences or tastes, due for instance to habit formation driving increased demand for fast foods. Whilst we cannot rule this out, we can investigate the extent to which it is likely to be an issue. To do this we use the rich information in the MCS, and examine whether conditional on individual FE and area‐level controls, the presence of fast food restaurants is correlated with time‐varying individual‐level characteristics. Table 5 provides evidence that they are generally not correlated with the presence of fast food restaurants around children's residences and schools.^20^

**TABLE 5:** Estimates of the effect of fast food restaurants around children's residences and schools on children and mother demographics.

	Two parents/carers	OECD equivalised weekly family income	NVQ level 4 or 5	Siblings of children in household	SDQ total score	General level of health
	(1)	(2)	(3)	(4)	(5)	(6)
Panel A: Homes						
Fast food restaurants within 1600 m	0.000234	−0.164	0.00111	0.00005	−0.00400	−0.00215
	(0.00239)	(0.674)	(0.00101)	(0.000707)	(0.0267)	(0.00520)
Fast food restaurants within 800 m	−0.00113	2.055	0.00252	−0.000722	−0.0595	−0.00560
	(0.00568)	(1.442)	(0.00204)	(0.00132)	(0.0528)	(0.0110)
Fast food restaurants within 400 m	0.000439	2.298	0.00567	−0.00144	−0.00280	−0.0549
	(0.00924)	(2.801)	(0.00468)	(0.00251)	(0.159)	(0.0342)
Panel B: Schools						
Fast food restaurants within 1600 m	0.00119	−1.074*	0.000319	−0.00002	−0.02740	−0.00007
	(0.00147)	(0.626)	(0.000764)	(0.000268)	(0.0198)	(0.00385)
Fast food restaurants within 800 m	−0.00205	−1.330	−0.000310	−0.000192	0.0309	0.00279
	(0.00483)	(1.399)	(0.00161)	(0.000395)	(0.0596)	(0.00929)
Fast food restaurants within 400 m	0.00484	0.882	0.00466	−0.00201	0.120	0.0330*
	(0.00713)	(2.602)	(0.00333)	(0.00180)	(0.0925)	(0.0172)
Observations	24,759	24,759	24,759	24,759	24,086	24,686
Children	8253	8253	8253	8253	8249	8253
Other food outlets	Yes	Yes	Yes	Yes	Yes	Yes
Individual controls	No	No	No	No	No	No
Area‐level economic controls	Yes	Yes	Yes	Yes	Yes	Yes
Individual FE	Yes	Yes	Yes	Yes	Yes	Yes
Year of survey FE	Yes	Yes	Yes	Yes	Yes	Yes

*Note*: This table shows OLS estimates. Each cell shows a different regression that includes a constant, other food outlets, area‐level economic controls, year of survey fixed effect, and individual fixed effect. Outcomes are shown at the top of each column. “Two parents/carers” is a dummy variable equals 1 if two parents or carers are present in the household and 0 otherwise; OECD equivalised weekly family income; “NVQ level 4 or 5” is a dummy variable equals 1 if the highest educational level of the mother is Degree or above level and 0 otherwise; the numbers of siblings of children in household; the individual's general health is reported by respondent's parent and ranges from 1 to 5, where 1 is excellent and 5 is poor health; and the Strengths & Difficulties Questionnaire (SDQ) total difficulties score. ***, **, and * denote statistically significant at 1%, 5% and 10%. Robust standard errors in parenthesis are clustered at the individual.

Another potential threat to our identification strategy is that families of certain sociodemographic characteristics may live disproportionately in areas with more fast food restaurants. It is difficult to completely rule out this concern without experimental data, but we provide evidence in the Appendix that it is unlikely to be an issue in our sample. We show our main effects are not driven by changes in fast food restaurants among those families that change residence (Tables A5 and A8). We also show that the presence of fast‐food restaurants in the buffer area close to where children live in period *t* is not associated with the probability of families changing residence between *t*−1 and *t* (Table A6). We additionally provide evidence that the number of fast‐food restaurants around children when they are aged 7 and 11 is not associated with future residential changes, between ages 7–11 and 11–14 respectively (Table A7). In the Appendix, we also provide evidence that our results are similar to estimates obtained with the estimator proposed by de Chaisemartin and D’Haultfœuille (2020), that allows for heterogeneity in treatment effects across time and treated units (Figures A5 and A6). Together with the fact that our results are robust to controlling for several time‐varying variables (including mode of transport from home to school in Table A9), these tests demonstrate that our estimates are unlikely to be biased by selective residential sorting or treatment effect heterogeneity.

We also show robustness of our results to changes in the classification of fast food restaurants (Tables A10 and A11). We modify our classification of fast food restaurants to include: Dixie Chicken, Chicken Cottage, Papa John's, Southern Fried Chicken, Five Guys, Harry Ramsdens and Little Chef. By including these additional fast food chains, our classification should resemble more closely the “Moderate” classification created by Wilkins et al. (2019a). Results in Tables A10 and A11 show that the magnitude and significance of estimated parameters remain stable.

Finally, we perform a placebo exercise by running our preferred specification but replacing our fast food variable with the number of facilities in other PoI categories that arguably should not be associated with childhood obesity. We use the categories “IT, marketing and media services,” “Employment and career agencies,” “Consultancies,” and “Construction services.”^21^ Figures A1 and A2 show the results for Equation (1), and Figures A3 and A4 for Equation (2). Across all specifications, we find no evidence that commercial facilities near children's residences or schools are associated with either BMI or body fat percentage, providing further evidence on the reliability of our main findings. This provides some evidence that our main results are not simply picking up time‐varying changes in unobserved area‐level characteristics, such as a gentrification of the neighborhood leading to a change in food social norms, economic activity, or food supply.

Another potential source of bias is measurement error in fast food restaurants classification around residences and schools, which would result in a conservative estimate of the effect of fast food restaurants on weight gain.

## 6 CONCLUSION

Using a nationally representative sample of young people across Great Britain, we show that increased exposure to fast food restaurants near homes and schools increases BMI during childhood and adolescence (ages 7–14). We find significant detrimental effects of exposure to fast food restaurants, but note that point estimates are relatively small, strikingly in line with previous evidence obtained on US data. Whilst the results are similar to previous studies, our findings are among the first for the UK. Our study improves on the existing evidence for the UK by using an identification strategy that controls for several confounders of the relationship between fast food restaurant availability and weight, such as residential sorting, fixed household heterogeneity, and time‐varying unobserved individual and area‐level factors. Although we cannot completely rule out self‐selection and omitted variable bias, we present evidence that our results are robust to different specifications and falsification tests.

A one standard deviation increase in fast food restaurants within 1600 m of individual residences (or 5.2 restaurants) increases BMI by 1.0% with respect to the sample mean, and by 0.5% within the 1600 m school‐buffer. Findings are similar for the other anthropometric measures that we examine. Our results also indicate that access to fast food restaurants surrounding schools is associated with increased weight, where a marginal increase in fast food restaurants within 800 m increases BMI and the incidence of overweight by 0.4% and 4.0% respectively with respect to the sample means. Comparing the findings for schools and homes, there is evidence that closer proximity to fast food restaurants—within 800 m—is detrimental for schools, whereas for homes, larger distances of up to 1600 m are more relevant. Although more research is needed to study whether this finding is echoed in other contexts, a potential explanation is that, as students gain independence, the purchase of takeaway fast foods may take place on the school journey.

Regarding heterogenous effects, the detrimental effects of fast food restaurants on BMI are larger among participants whose mothers have relatively low levels of education. These results are consistent with educational inequalities within the household amplifying health inequalities at early ages (Deaton, 2003; Marmot, 2010) and with cross‐sectional evidence in the UK, which shows that access to fast food restaurants accentuates socioeconomic inequalities in adults (Burgoine et al., 2016). The inter‐relationship between nutrition knowledge and maternal education is one potential explanation for the larger estimated effect of fast food exposure among those with poorer socioeconomic conditions (Parmenter et al., 2000). Yet, an alternative explanation may be that time and budget constraints are more binding among disadvantaged families, who may have less time to prepare meals and are less able to afford nutritious foods which are typically more expensive than processed foods.

We examine heterogeneity with respect to emotional regulation, finding that the negative effects of fast food proximity are significantly larger among participants with lower levels of emotional regulation compared to those with higher levels. Combined with our findings that those from more disadvantaged backgrounds are at heightened risk from the effects of fast food supply, this suggests that more attention be paid to planning decisions in less advantaged areas. Finally, we find no evidence that the low estimated impacts reflect compensatory behaviors, including changes in dietary quality and physical activity.

We have focused specifically in this research on the role played by growing up in an obesogenic environment in contributing to increased childhood obesity. We fully recognize that the surge in childhood obesity is likely driven by a myriad of socioeconomic, cultural and environmental factors, alongside interactions between them and more structural ones such as those examined in this paper. Understanding the interplay between these factors remains a key area for future research to inform policy to tackle health inequalities.

## CONFLICT OF INTEREST STATEMENT

The authors declare no conflicts of interest.

## ETHICS STATEMENT

The UK Millennium Cohort Study has received ethical approval from the National Health Service (NHS) Research Ethics Committee (REC) system. Ethical approval has been sought for all MCS surveys since the start of the study in 1999.

## Supporting information

Supporting Information S1

## Data Availability

The Millennium Cohort Study data supporting this study's findings are openly available in UK Data Service at beta.ukdataservice.ac.uk/datacatalogue/series/series?id=2000031. The Ordnance Survey Point of Interest Dataset supporting this study's findings is available from the Centre for Longitudinal Studies, University College London. Restrictions apply to the availability of these data, which were used under license for this study.

## ENDNOTES

^1^
In 2018, the BBC Shared Data Unit reported a 34% increase in fast food outlets from 2010 to 2018 in the UK, and an increase in the average number of fast food outlets per 100,000 people from 47 in 2010 to 61 by 2018 (BBC, 2018) Figures from Public Health England (PHE) reveal England's poorest areas have 5 times more fast food outlets than the most affluent (PHE, 2018).

^2^
Food and drink provided for purchase to pupils at schools must comply with certain nutritional standards, designed to help children develop have healthy and balanced diets. Free school meals are available to all infants (approximately ages 4–7), and to families in receipt of certain household benefits. Students can also take packed lunches to school if they choose to.

^3^
The MCS 17 survey data was not available at the time of conducting the analysis.

^4^
In the Appendix we show results for BMI standardized scores, percentage of body fat, weight, overweight and obese. We standardized respondent's BMI by age and sex using the 1990 UK Growth Reference (Cole et al., 1995).

^5^
Respondents' weight and height have been measured in the home by trained interviewers. A Leicester height stadiometer is a foldable device with a sliding head plate, a base plate and four connecting rods marked with a measuring scale.

^6^
In the Appendix we show the PoI categories used in this study.

^7^
Due to a change of supplier in late 2010, which resulted in improved data collection and classification methods, there is some difference in raw category counts in pre‐ and post‐2010 PoI data. For example, Cummins et al. (2005) used the Yellow Pages to identify every listing for McDonald's, finding 942 in January 2005. In the September 2005 PoI dataset, there are 850 McDonald's outlets.

^8^
ITN data are available from Edina Digimap on a non‐regular basis ‐i.e. 1–3 times per year‐from February 2007.

^9^
Postcodes are the smallest geographical administrative area in the UK. There are around 1.8 million postcodes, and they could contain between 1 and 300 addresses with an average of 15 addresses per postcode. Since schools generally comprise a single unit postcode, school locations are generally located more accurately than homes.

^10^
For instance, when we consider the following list of fast food chains: McDonalds, KFC, Burger King, Wimpy, Subway, Pizza Hut, and Dominos' Pizza, we find that 25.9% (*n* = 619) of fast food chains around 1600 m from respondent's residence at age 7 were not included in categories “Fast food and takeaway outlets” or “Fast food and delivery services.”

^11^
Wilkins et al. (2019a) evaluated three classifications of fast food outlets: Narrow, Moderate and Broad. In their Narrow classification, they included, in addition to these major chains, the following outlets: Dixie Chicken, Chicken Cottage, Papa John's, Southern Fried Chicken (SFC), Five Guys, Harry Ramsdens, and Little Chef. Their Moderate classification adds outlets serving burgers, kebabs, fried chicken, fish and chips, pizza, Indian and Chinese outlets. Their Broad classification further adds takeaway cafes, retail bakeries and chain coffee shops (e.g., Starbucks). Our classification is closer to the Moderate classification in Wilkins et al. (2019a). It is found to be provide major agreements in statistical significance of finding in the cross‐sectional association with BMI than the Narrow classification of fast food outlets. We show in Tables A10 and A11 that our results are not driven by changes in the classification of fast food outlets.

^12^
The education level categories correspond to none, National Vocational Qualification (NVQ) levels from 1 to 5, and overseas qualification only. NVQ levels rank an individual's qualification by difficulty. For example, NVQ 1–3 levels include different levels of High School certificates and qualifications; NVQ 4 level includes bachelor's degrees, graduate certificates, and other higher education diplomas; and NVQ 5 level includes master's degrees, Postgraduate certificates/diplomas and Doctorate degrees.

^13^
Local authority districts are sub‐national areas used for purposes of local governments in the UK. There are 379 local authority districts in the UK, with a population average in 2016 of around 173,000 people ranging from 2300 to 1,128,000 people.

^14^
In the Appendix we describe the other food outlets used in our main specification.

^15^
Overweight and obesity were constructed using International Obesity Task Force (IOTF) BMI cut‐off points (Cole et al., 2000) and derived using sex and age to the nearest 10th of the year.

^16^
Note that comparing point estimates between studies is challenging as we are not using the same methods in the identification strategy; however, the comparison is useful to evaluate the magnitude of our point estimates.

^17^
Our measures of consumption captures low fruit consumption, consumption of sweetened drinks and skipping breakfast. Low fruit consumption is a binary variable indicating whether the individual does not eats fruits. Consumption of sweetened drinks is a binary variable defined as whether the individual drinks sweetened drinks. Skipping breakfast is a binary variable indicating whether the individual skips breakfast at least once a week.

^18^
Following our analysis on heterogenous effects described in Section 4.4, we also estimate impacts after stratifying the sample by low/high maternal education, and low/high emotional regulation, and find no impacts on markers of healthy diet for these sub‐groups (estimates are available upon request).

^19^
Estimates are available upon request.

^20^
Only two of 36 coefficients are significant at 10% level.

^21^
In Table B2 we show a description of the facilities consider in each of these four categories.
