# Supplementary material for: Fast food proximity and weight gain in childhood and adolescence: Evidence from Great Britain
Source: Health Econ. 2023 Nov 16;33(3):449–65. doi: 10.1002/hec.4770 (PMC10952272; doi:10.1002/hec.4770)
Supplement: Supplementary file 1 — Supporting Information S1 [file HEC-33-449-s001.docx]

**ONLINE APPENDIX**

**Appendix A**

## Identification strategy

## Residential sorting

As stated in the main text, our empirical specification identifies the impact of fast food restaurants on BMI under the assumption that, conditional on individual and year of survey fixed effects, and controlling for local authority district and individual time-varying characteristics, the within-individual variation in fast food outlets over time is independent of other determinants of BMI.

One reason why the supply of fast food restaurants near the individuals’ homes (or schools) might change is individuals moving residence (or school) to or from areas with more or fewer fast food restaurants. Whilst it is very unlikely that families are making their residential decisions on the basis of fast food presence, the fact that fast food supply is likely correlated with other local amenities relevant to residential decisions, such as the quality schools, is an important consideration. We explore this empirically in the following ways. First, we estimate equation (1) but interact both food outlet variables with a binary variable indicating whether families did not change residence during the period. We do not find a differential effect on the impact of fast food restaurants on BMI between those families who changed residence and those who did not (Table A5). This provides suggestive evidence that our results are not driven by changes in exposure to fast food restaurants among those families that changed residence during this period. Second, to formally test whether the number of fast food restaurants at age t= 7, 11 and 14 is correlated with the probability of individuals changing residence between t-1and t, we estimate the following model:

$D_{it}=\tau^{k}+\theta^{k}F_{it}^{k}+\nu_{i}^{k}+\mu_{t}^{k}+\epsilon_{it}^{k}$ (3)

where $D_{it}$ is a binary variable indicating if individual changed residence between t-1 and t, considering changes of residences between ages 5-7, 7-11 and 11-14 years. $\tau^{k}$ is a constant term, $\nu_{i}^{k}$ is an individual fixed effect, and $\mu_{t}^{k}$ is a year of the survey dummy variable. The superscript k indicates buffers of 400, 800, and 1,600 metres around residences and schools. Results shown in Table A6 show a null association between the number of fast food restaurants in t and the probability of changing residence between t-1 and t.

$D_{it}=\alpha^{k}+\eta^{k}F_{it-1}^{k}+\nu_{i}^{k}+\mu_{t}^{k}+\epsilon_{it}^{k}$ (4)

We also estimate equation (4) and evaluate whether the number of fast food restaurants at ages 7 and 11 is correlated with changes of residence between ages 7-11 and 11-14. The results reported in Table A7 show null effects. We interpret this evidence as indicating that families within our sample are not deciding where to move based on fast food supply.

Third, as a robustness check, we re-estimate equations (1) and (2) but controlling for $D_{it}$ to assess whether our estimates change. If $D_{it}$ captures time-varying family unobservables correlated with the decision to change residence, our finding that estimates remain stable after controlling for $D_{it}$ – shown in Table A8 – provides evidence that residential sorting is not driving our results.

## TWFE models under heterogeneity in treatment effects across time and treated units

Two Way Fixed Effects (TWFE) models deliver consistent estimates of the Average Treatment on Treated (ATT) only under relatively strong assumptions about homogeneity in treatment effects ([Borusyak, Jaravel, & Spiess, 2021](#_ENREF_2); [Callaway & Sant’Anna, 2021](#_ENREF_3); [Goodman-Bacon, 2021](#_ENREF_7); [Sun & Abraham, 2021](#_ENREF_10)). The TWFE does not deliver a consistent estimate for the ATT if treatment effects are heterogenous across time and individuals. [de Chaisemartin and D’Haultfœuille (2020)](#_ENREF_5) proposed an estimator, DID_M_, that allows for heterogeneity in treatment effects across time and treated units even when the treatment is non-binary, which is the case in our study, i.e., the number of fast food restaurants near 1600 meters around individual residences and schools.

We address concerns about the reliability of our TWFE estimator by replicating our main results using the DID_M_, which estimates the average treatment effect across all time and units cells where the number of fast food outlets changes from t-1 to t. In Figures A5 and A6 we show results of the effect of proximity to fast food restaurants within 1600 buffers around residences and schools on BMI, z standardized scores of BMI, percentage of body fat and weight. Overall, our results for these four continuous variables are similar or larger than our main results, indicating that the estimates presented in the paper are likely conservative estimates.

Table A1. Presence of fast food restaurants and other food outlets around individual’s home and across time

|  | (1) | (2) | (3) | (4) | (5) | (6) | (7) | (8) | (9) |
| --- | --- | --- | --- | --- | --- | --- | --- | --- | --- |
|  | 1600 metres | | | 800 metres | | | 400 metres | | |
|  | 7 years | 11 years | 14 years | 7 years | 11 years | 14 years | 7 years | 11 years | 14 years |
| Fast Food Restaurants |  |  |  |  |  |  |  |  |  |
| Fast Food | 2.54 | 4.10 | 3.97 | 0.63 | 1.04 | 1.00 | 0.15 | 0.25 | 0.23 |
| McDonalds | 0.12 | 0.18 | 0.16 | 0.02 | 0.03 | 0.03 | 0.00 | 0.00 | 0.00 |
| KFC | 0.11 | 0.14 | 0.14 | 0.02 | 0.02 | 0.02 | 0.00 | 0.00 | 0.00 |
| Burger King | 0.03 | 0.04 | 0.04 | 0.00 | 0.00 | 0.00 | 0.00 | 0.00 | 0.00 |
| Wimpy | 0.02 | 0.03 | 0.02 | 0.00 | 0.00 | 0.00 | 0.00 | 0.00 | 0.00 |
| Subway | 0.07 | 0.20 | 0.24 | 0.01 | 0.04 | 0.05 | 0.00 | 0.01 | 0.01 |
| Pizza Hut | 0.09 | 0.16 | 0.11 | 0.01 | 0.03 | 0.02 | 0.00 | 0.01 | 0.00 |
| Domin’'s Pizza | 0.08 | 0.25 | 0.16 | 0.01 | 0.05 | 0.03 | 0.00 | 0.01 | 0.01 |
| Kebab & Chicken | 0.65 | 1.31 | 1.31 | 0.16 | 0.32 | 0.33 | 0.03 | 0.08 | 0.07 |
| Fish and chip shops | 1.36 | 1.80 | 1.80 | 0.40 | 0.54 | 0.53 | 0.11 | 0.14 | 0.14 |
| Others Food Facilities |  |  |  |  |  |  |  |  |  |
| Other Food Outlets | 20.07 | 29.75 | 29.43 | 5.20 | 7.80 | 7.47 | 1.29 | 1.89 | 1.80 |
| Restaurants | 4.18 | 5.98 | 6.23 | 0.96 | 1.43 | 1.43 | 0.21 | 0.31 | 0.30 |
| Bakeries | 0.95 | 1.27 | 1.27 | 0.24 | 0.34 | 0.32 | 0.06 | 0.08 | 0.07 |
| Butchers | 1.10 | 1.28 | 1.19 | 0.30 | 0.35 | 0.29 | 0.08 | 0.09 | 0.08 |
| Confectioners | 0.21 | 0.44 | 0.42 | 0.05 | 0.11 | 0.10 | 0.01 | 0.02 | 0.03 |
| Delicatessens | 0.28 | 0.57 | 0.59 | 0.06 | 0.14 | 0.15 | 0.01 | 0.03 | 0.03 |
| Fishmongers | 0.10 | 0.19 | 0.16 | 0.02 | 0.05 | 0.04 | 0.00 | 0.01 | 0.01 |
| Green and new age goods | 0.01 | 0.04 | 0.03 | 0.00 | 0.01 | 0.01 | 0.00 | 0.00 | 0.00 |
| Grocers; farm shops and pick your own | 0.89 | 1.75 | 1.50 | 0.25 | 0.45 | 0.37 | 0.06 | 0.11 | 0.09 |
| Organic; health; gourmet and kosher foods | 0.38 | 0.32 | 0.38 | 0.09 | 0.06 | 0.08 | 0.02 | 0.01 | 0.01 |
| Conv. stores and independent supermarkets | 5.38 | 9.43 | 9.21 | 1.52 | 2.60 | 2.49 | 0.42 | 0.68 | 0.65 |
| Supermarket chains | 1.56 | 1.55 | 1.40 | 0.37 | 0.42 | 0.36 | 0.09 | 0.11 | 0.09 |
| Other takeaway outlets | 5.02 | 6.92 | 7.04 | 1.33 | 1.84 | 1.82 | 0.32 | 0.44 | 0.43 |
| Observations | 8253 | 8253 | 8253 | 8253 | 8253 | 8253 | 8253 | 8253 | 8253 |

Note: This table shows descriptive statistics of variables used to create our ‘fast food restaurants’ and ‘other food facilities’ we use in the regression analysis.

Table A2. Presence of fast food restaurants and other food outlets around individual’s school and across time

|  | (1) | (2) | (3) | (4) | (5) | (6) | (7) | (8) | (9) |
| --- | --- | --- | --- | --- | --- | --- | --- | --- | --- |
|  | 1600 metres | | | 800 metres | | | 400 metres | | |
|  | 7 years | 11 years | 14 years | 7 years | 11 years | 14 years | 7 years | 11 years | 14 years |
| Fast Food Restaurants |  |  |  |  |  |  |  |  |  |
| Fast Food | 2.69 | 3.91 | 3.80 | 0.75 | 1.08 | 0.84 | 0.20 | 0.20 | 0.15 |
| McDonalds | 0.14 | 0.17 | 0.15 | 0.02 | 0.04 | 0.02 | 0.00 | 0.01 | 0.01 |
| KFC | 0.11 | 0.12 | 0.11 | 0.02 | 0.03 | 0.02 | 0.00 | 0.00 | 0.00 |
| Burger King | 0.03 | 0.05 | 0.05 | 0.00 | 0.02 | 0.01 | 0.00 | 0.01 | 0.00 |
| Wimpy | 0.02 | 0.03 | 0.02 | 0.00 | 0.02 | 0.01 | 0.00 | 0.00 | 0.00 |
| Subway | 0.08 | 0.23 | 0.28 | 0.01 | 0.07 | 0.07 | 0.00 | 0.01 | 0.01 |
| Pizza Hut | 0.10 | 0.14 | 0.10 | 0.02 | 0.03 | 0.02 | 0.00 | 0.01 | 0.00 |
| Domino's Pizza | 0.09 | 0.30 | 0.17 | 0.02 | 0.07 | 0.03 | 0.01 | 0.01 | 0.01 |
| Kebab & Chicken | 0.64 | 1.10 | 1.10 | 0.17 | 0.29 | 0.22 | 0.03 | 0.05 | 0.04 |
| Fish and chip shops | 1.48 | 1.79 | 1.82 | 0.49 | 0.53 | 0.44 | 0.15 | 0.11 | 0.07 |
| Others Food Facilities |  |  |  |  |  |  |  |  |  |
| Other Food Outlets | 21.53 | 29.83 | 30.30 | 6.04 | 7.95 | 6.80 | 1.60 | 1.40 | 1.29 |
| Restaurants | 4.73 | 6.45 | 6.86 | 1.13 | 1.70 | 1.37 | 0.26 | 0.22 | 0.24 |
| Bakeries | 1.02 | 1.43 | 1.44 | 0.31 | 0.38 | 0.34 | 0.08 | 0.05 | 0.06 |
| Butchers | 1.16 | 1.24 | 1.17 | 0.38 | 0.33 | 0.26 | 0.11 | 0.06 | 0.04 |
| Confectioners | 0.23 | 0.45 | 0.49 | 0.06 | 0.12 | 0.12 | 0.01 | 0.02 | 0.03 |
| Delicatessens | 0.33 | 0.62 | 0.62 | 0.09 | 0.16 | 0.14 | 0.02 | 0.03 | 0.03 |
| Fishmongers | 0.10 | 0.20 | 0.14 | 0.03 | 0.04 | 0.02 | 0.00 | 0.00 | 0.00 |
| Green and new age goods | 0.02 | 0.04 | 0.03 | 0.00 | 0.01 | 0.01 | 0.00 | 0.00 | 0.00 |
| Grocers; farm shops and pick your own | 0.95 | 1.50 | 1.29 | 0.27 | 0.33 | 0.26 | 0.07 | 0.06 | 0.05 |
| Organic; health; gourmet and kosher foods | 0.43 | 0.42 | 0.43 | 0.12 | 0.11 | 0.09 | 0.03 | 0.02 | 0.02 |
| Conv. stores and independent supermarkets | 5.63 | 8.80 | 8.86 | 1.68 | 2.39 | 2.04 | 0.50 | 0.51 | 0.43 |
| Supermarket chains | 1.64 | 1.69 | 1.61 | 0.45 | 0.46 | 0.39 | 0.12 | 0.09 | 0.07 |
| Other takeaway outlets | 5.28 | 6.99 | 7.34 | 1.51 | 1.92 | 1.75 | 0.39 | 0.33 | 0.32 |
| Observations | 8253 | 8253 | 8253 | 8253 | 8253 | 8253 | 8253 | 8253 | 8253 |

Note: This table shows descriptive statistics of variables used to create our ‘fast food restaurants’ and ‘other food facilities’ we use in the regression analysis.

Table A3. The impact of fast food restaurants on respondents’ anthropometric measurements, cross-sectional estimates.

|  | Home | | | |  | | | | School | | | |
| --- | --- | --- | --- | --- | --- | --- | --- | --- | --- | --- | --- | --- |
|  | BMI | Body fat (%) | Weight (Kg.) | z-BMI | Overweight | Obese | BMI | Body fat (%) | Weight (Kg.) | z-BMI | Overweight | Obese |
|  | (1) | (2) | (3) | (4) | (5) | (6) | (7) | (8) | (9) | (10) | (11) | (12) |
| Panel A. Fast food restaurants within |  |  |  |  |  |  |  |  |  |  |  |  |
| Equation 1, k = 400m | 0.142** | 0.326** | 0.228 | 0.0462* | 1.726* | 0.586 | 0.150** | 0.196 | 0.347** | 0.0547*** | 2.131** | 1.173** |
|  | (0.0654) | (0.141) | (0.170) | (0.0245) | (0.962) | (0.457) | (0.0626) | (0.125) | (0.175) | (0.0200) | (0.843) | (0.553) |
| Equation 1, k = 800m | 0.0895*** | 0.168** | 0.208** | 0.0324*** | 0.779** | 0.277 | 0.113*** | 0.203*** | 0.224** | 0.0348*** | 0.944*** | 0.693*** |
|  | (0.0331) | (0.0651) | (0.0910) | (0.0116) | (0.376) | (0.213) | (0.0260) | (0.0515) | (0.0869) | (0.00884) | (0.332) | (0.180) |
| Equation 1, k = 1600m | 0.0568*** | 0.106*** | 0.137*** | 0.0187*** | 0.428** | 0.303*** | 0.0339** | 0.0779*** | 0.0335 | 0.00958** | 0.252 | 0.284*** |
|  | (0.0181) | (0.0368) | (0.0496) | (0.00631) | (0.188) | (0.109) | (0.0131) | (0.0288) | (0.0346) | (0.00445) | (0.178) | (0.0906) |
| Panel B. Equation 2 |  |  |  |  |  |  |  |  |  |  |  |  |
| Fast food restaurants |  |  |  |  |  |  |  |  |  |  |  |  |
| within 400m | 0.131** | 0.304** | 0.202 | 0.0440* | 1.647* | 0.461 | 0.149** | 0.192 | 0.345* | 0.0546*** | 2.102** | 1.129** |
|  | (0.0649) | (0.141) | (0.170) | (0.0243) | (0.969) | (0.452) | (0.0622) | (0.124) | (0.176) | (0.0200) | (0.839) | (0.545) |
| between 400m and 800m | 0.0631* | 0.104 | 0.170* | 0.0255** | 0.451 | 0.0648 | 0.107*** | 0.202*** | 0.213** | 0.0321*** | 0.730** | 0.531*** |
|  | (0.0340) | (0.0751) | (0.102) | (0.0116) | (0.415) | (0.219) | (0.0287) | (0.0579) | (0.0953) | (0.00960) | (0.339) | (0.196) |
| between 800m and 1600m | 0.0500** | 0.0920** | 0.125** | 0.0153** | 0.339 | 0.346*** | 0.00894 | 0.0397 | -0.0295 | 0.00137 | 0.0353 | 0.175 |
|  | (0.0210) | (0.0427) | (0.0613) | (0.00729) | (0.223) | (0.127) | (0.0148) | (0.0331) | (0.0391) | (0.00501) | (0.199) | (0.110) |
|  |  |  |  |  |  |  |  |  |  |  |  |  |
| Other food outlets | Yes | Yes | Yes | Yes | Yes | Yes | Yes | Yes | Yes | Yes | Yes | Yes |
| Individual controls | Yes | Yes | Yes | Yes | Yes | Yes | Yes | Yes | Yes | Yes | Yes | Yes |
| Area level controls | Yes | Yes | Yes | Yes | Yes | Yes | Yes | Yes | Yes | Yes | Yes | Yes |
| Year of survey FE | Yes | Yes | Yes | Yes | Yes | Yes | Yes | Yes | Yes | Yes | Yes | Yes |
| Observations | 24,759 | 24,466 | 24,759 | 24,759 | 24,759 | 24,748 | 24,759 | 24,466 | 24,759 | 24,759 | 24,759 | 24,748 |
| Mean of dependent variable | 18.95 | 21.56 | 41.36 | 23.05 | 5.784 | 0.433 | 18.95 | 21.56 | 41.36 | 23.05 | 5.784 | 0.433 |

Notes: This table show OLS estimates for cross-sectional models. Columns 1-6 show estimates around respondents’ residences and columns 7-12 around schools. In Panel A, each cell reports a different regression, and rows show results for three different equations – one for each respective buffer in equation (1): $\beta^{400}$, $\beta^{800}$ , and $\beta^{1600}$. In Panel B, rows show estimates of equation (2): $\gamma_{1}$,$\gamma_{2}$, and $\gamma_{3}$. Other estimates in equations (1) and (2) are omitted due to space restrictions but available upon request. ***, **, and * denote statistically significant at 1%, 5% and 10%. Robust standard errors in parenthesis are clustered at the individual level.

Table A4. The impact of fast food restaurants on respondents’ anthropometric measurements, fixed effects estimates.

|  | Home | | | | | School | | | | |
| --- | --- | --- | --- | --- | --- | --- | --- | --- | --- | --- |
|  | Body fat (%) | Weight (Kg.) | z-BMI | Overweight | Obese | Body fat (%) | Weight (Kg.) | z-BMI | Overweight | Obese |
|  | (1) | (2) | (3) | (4) | (5) | (6) | (7) | (8) | (9) | (10) |
| Panel A. Equation 1 |  |  |  |  |  |  |  |  |  |  |
| Fast food restaurants within 400m | 0.252 | 0.344 | 0.0288 | 1.608 | -0.0125 | -0.0375 | -0.0700 | 0.0124 | 1.499** | 0.846** |
|  | (0.183) | (0.214) | (0.0289) | (1.293) | (0.552) | (0.104) | (0.130) | (0.0130) | (0.729) | (0.395) |
| Fast food restaurants within 800m | 0.121* | 0.157** | 0.0175** | 0.537 | 0.0150 | 0.112* | 0.167** | 0.0223*** | 0.955** | 0.308 |
|  | (0.0675) | (0.0776) | (0.00891) | (0.364) | (0.233) | (0.0586) | (0.0718) | (0.00752) | (0.371) | (0.202) |
| Fast food restaurants within 1600m | 0.0849** | 0.0858** | 0.00798* | 0.251 | 0.0711 | 0.0180 | 0.0304 | 0.00432 | 0.184 | 0.226** |
|  | (0.0343) | (0.0380) | (0.00425) | (0.189) | (0.130) | (0.0253) | (0.0315) | (0.00339) | (0.137) | (0.103) |
| Panel B. Equation 2 |  |  |  |  |  |  |  |  |  |  |
| Fast food restaurants |  |  |  |  |  |  |  |  |  |  |
| within 400m | 0.244 | 0.359* | 0.0311 | 1.651 | -0.0314 | -0.0230 | -0.0525 | 0.0153 | 1.590** | 0.876** |
|  | (0.183) | (0.217) | (0.0284) | (1.261) | (0.553) | (0.104) | (0.131) | (0.0129) | (0.721) | (0.396) |
| between 400m and 800m | 0.0957 | 0.106 | 0.0138 | 0.277 | -0.00726 | 0.140** | 0.211*** | 0.0236*** | 0.821** | 0.188 |
|  | (0.0810) | (0.0936) | (0.0105) | (0.493) | (0.246) | (0.0615) | (0.0744) | (0.00811) | (0.387) | (0.227) |
| between 800m and 1600m | 0.0698* | 0.0604 | 0.00467 | 0.144 | 0.0885 | -0.0111 | -0.0118 | -0.000886 | -0.0386 | 0.203* |
|  | (0.0370) | (0.0427) | (0.00447) | (0.216) | (0.145) | (0.0285) | (0.0371) | (0.00373) | (0.165) | (0.108) |
|  |  |  |  |  |  |  |  |  |  |  |
| Other food outlets | Yes | Yes | Yes | Yes | Yes | Yes | Yes | Yes | Yes | Yes |
| Individual controls | Yes | Yes | Yes | Yes | Yes | Yes | Yes | Yes | Yes | Yes |
| Area level controls | Yes | Yes | Yes | Yes | Yes | Yes | Yes | Yes | Yes | Yes |
| Individual FE | Yes | Yes | Yes | Yes | Yes | Yes | Yes | Yes | Yes | Yes |
| Year of survey FE | Yes | Yes | Yes | Yes | Yes | Yes | Yes | Yes | Yes | Yes |
| Observations | 24,466 | 24,759 | 24,748 | 24,759 | 24,759 | 24,466 | 24,759 | 24,748 | 24,759 | 24,759 |
| Number of individuals | 8,252 | 8,253 | 8,253 | 8,253 | 8,253 | 8,252 | 8,253 | 8,253 | 8,253 | 8,253 |
| Mean of dependent variable | 21.56 | 41.36 | 0.433 | 23.05 | 5.784 | 21.56 | 41.36 | 0.433 | 23.05 | 5.784 |

Notes: This table show OLS estimates for fixed-effect models. Columns 1-6 show estimates around respondents’ residences and columns 7-12 around schools. In Panel A, each cell reports a different regression, and rows show results for three different equations – one for each respective buffer in equation (1): $\beta^{400}$, $\beta^{800}$ , and $\beta^{1600}$. In Panel B, rows show estimates of equation (2): $\gamma_{1}$,$\gamma_{2}$, and $\gamma_{3}$. Other estimates in equations (1) and (2) are omitted due to space restrictions but available upon request. Overweight and obesity were constructed using IOTF BMI cut-off points and derived using sex and age to the nearest 10^th^ of the year. z-BMI are BMI standardized scores derived using sex, age, and the 1990 UK Growth Reference. ***, **, and * denote statistically significant at 1%, 5% and 10%. Robust standard errors in parenthesis are clustered at the individual level.

Table A5. Differential effects by change of residence

|  | Home | | | School | | |
| --- | --- | --- | --- | --- | --- | --- |
|  | (1) | (2) | (3) | (4) | (5) | (6) |
| Panel A. Equation 1 |  |  |  |  |  |  |
| Fast food restaurants within 400m | 0.185 |  |  | -0.0913 |  |  |
|  | (0.121) |  |  | (0.0833) |  |  |
| Fast food restaurants within 400m x Stay | -0.209 |  |  | 0.179* |  |  |
|  | (0.138) |  |  | (0.0951) |  |  |
| Fast food restaurants within 800m |  | 0.0853** |  |  | 0.104** |  |
|  |  | (0.0389) |  |  | (0.0439) |  |
| Fast food restaurants within 800m x Stay |  | -0.0605 |  |  | -0.0467 |  |
|  |  | (0.0506) |  |  | (0.0499) |  |
| Fast food restaurants within 1600m |  |  | 0.0363* |  |  | 0.0270 |
|  |  |  | (0.0192) |  |  | (0.0195) |
| Fast food restaurants within 1600m x Stay |  |  | -0.000864 |  |  | -0.00708 |
|  |  |  | (0.0247) |  |  | (0.0233) |
| Other food outlets | Yes | Yes | Yes | Yes | Yes | Yes |
| Individual controls | Yes | Yes | Yes | Yes | Yes | Yes |
| Area level controls | Yes | Yes | Yes | Yes | Yes | Yes |
| Year of survey FE | Yes | Yes | Yes | Yes | Yes | Yes |
| Observations | 24,759 | 24,759 | 24,759 | 24,759 | 24,759 | 24,759 |
| Number of individuals | 8253 | 8253 | 8253 | 8253 | 8253 | 8253 |
| Mean of dependent variable | 18.95 | 18.95 | 18.95 | 18.95 | 18.95 | 18.95 |

Notes: This table show OLS results for a modified equation (1) that interact the fast food restaurants and other food outlets variables with $D_{it}$, named Stay. $D_{it}$ is a dummy variable that takes value 1 if respondents changed residence between t-1 and t, i.e. between ages 5-7, 7-11 and 11-14. ***, **, and * denote statistically significant at 1%, 5% and 10%. Robust standard errors in parenthesis are clustered at the individual level.

Table A6. Estimates of the probability of moving residence between t-1 and t as function of fast food restaurants in t.

|  | Home | | | School | | |
| --- | --- | --- | --- | --- | --- | --- |
|  | (1) | (2) | (3) | (4) | (5) | (6) |
|  |  |  |  |  |  |  |
| Fast food restaurants within 1600m | -0.00136 |  |  |  |  |  |
|  | (0.00196) |  |  |  |  |  |
| Fast food restaurants within 800m |  | 0.00416 |  |  |  |  |
|  |  | (0.00467) |  |  |  |  |
| Fast food restaurants within 400m |  |  | 0.00419 |  |  |  |
|  |  |  | (0.0101) |  |  |  |
| Fast food restaurants within 1600m |  |  |  | 0.000852 |  |  |
|  |  |  |  | (0.00179) |  |  |
| Fast food restaurants within 800m |  |  |  |  | -0.000770 |  |
|  |  |  |  |  | (0.00308) |  |
| Fast food restaurants within 400m |  |  |  |  |  | -0.00670 |
|  |  |  |  |  |  | (0.00713) |
| Survey year = 5 (age 11) | 0.0571*** | 0.0533*** | 0.0546*** | 0.0540*** | 0.0553*** | 0.0550*** |
|  | (0.00817) | (0.00794) | (0.00782) | (0.00765) | (0.00769) | (0.00776) |
| Survey year = 6 (age 14) | -0.00102 | -0.00452 | -0.00328 | -0.00392 | -0.00290 | -0.00333 |
|  | (0.00790) | (0.00749) | (0.00727) | (0.00743) | (0.00721) | (0.00723) |
| Constant | 0.185*** | 0.179*** | 0.181*** | 0.180*** | 0.182*** | 0.183*** |
|  | (0.00658) | (0.00519) | (0.00454) | (0.00683) | (0.00507) | (0.00453) |
|  |  |  |  |  |  |  |
| Other Food Outlets | No | No | No | No | No | No |
| Individual controls | No | No | No | No | No | No |
| Area level economic controls | No | No | No | No | No | No |
| Individual FE | Yes | Yes | Yes | Yes | Yes | Yes |
| Year of survey FE | Yes | Yes | Yes | Yes | Yes | Yes |
| Observations | 24,759 | 24,759 | 24,759 | 24,759 | 24,759 | 24,759 |
| Number of individuals | 8,253 | 8,253 | 8,253 | 8,253 | 8,253 | 8,253 |

Notes: This table shows estimates for equation (3) $D_{it}=\tau^{k}+\theta^{k}F_{it}^{k}+\nu_{i}^{k}+\mu_{t}^{k}+\epsilon_{it}^{k}$. The outcome is a binary variable indicating if respondents changed residence between ages t-1 and t, i.e., between ages 5-7, 7-11 and 11-14. The variable fast food restaurants in the buffer k ($F_{it}^{k}$) is measured at ages 7, 11 and 14. ***, **, and * denote statistically significant at 1%, 5% and 10%. Robust standard errors in parenthesis are clustered at the individual level.

Table A7. Estimates of the probability of moving residence between t-1 and t as function of fast food restaurants in t-1.

|  | Home | | | School | | |
| --- | --- | --- | --- | --- | --- | --- |
|  | (1) | (2) | (3) | (4) | (5) | (6) |
|  |  |  |  |  |  |  |
| Fast food restaurants within 400m |  |  | -0.00325 |  |  |  |
|  |  |  | (0.0186) |  |  |  |
| Fast food restaurants within 800m |  | 0.00180 |  |  |  |  |
|  |  | (0.00690) |  |  |  |  |
| Fast food restaurants within 1600m | 0.00357 |  |  |  |  |  |
|  | (0.00322) |  |  |  |  |  |
| Fast food restaurants within 400m |  |  |  |  |  | 0.000788 |
|  |  |  |  |  |  | (0.00894) |
| Fast food restaurants within 800m |  |  |  |  | 0.00517 |  |
|  |  |  |  |  | (0.00350) |  |
| Fast food restaurants within 1600m |  |  |  | -0.00126 |  |  |
|  |  |  |  | (0.00213) |  |  |
| Survey year = 6 (age 14) | -0.0636*** | -0.0587*** | -0.0577*** | -0.0565*** | -0.0597*** | -0.0580*** |
|  | (0.00854) | (0.00784) | (0.00765) | (0.00766) | (0.00759) | (0.00761) |
| Constant | 0.228*** | 0.236*** | 0.237*** | 0.240*** | 0.233*** | 0.237*** |
|  | (0.00949) | (0.00608) | (0.00496) | (0.00733) | (0.00483) | (0.00398) |
|  |  |  |  |  |  |  |
| Other Food Outlets | No | No | No | No | No | No |
| Individual controls | No | No | No | No | No | No |
| Area level economic controls | No | No | No | No | No | No |
| Individual FE | Yes | Yes | Yes | Yes | Yes | Yes |
| Year of survey FE | Yes | Yes | Yes | Yes | Yes | Yes |
| Observations | 16,506 | 16,506 | 16,506 | 16,506 | 16,506 | 16,506 |
| Number of individuals | 8,253 | 8,253 | 8,253 | 8,253 | 8,253 | 8,253 |

Notes: This table shows estimates for equation (4) $D_{it}=\alpha^{k}+\eta^{k}F_{it-1}^{k}+\nu_{i}^{k}+\mu_{t}^{k}+\epsilon_{it}^{k}$ . The outcome is a binary variable indicating if respondents changed residence between ages 7-11 and 11-14 years. The variable fast food restaurants ($F_{it-1}^{k}$) is measured at ages 7 and 11. ***, **, and * denote statistically significant at 1%, 5% and 10%. Robust standard errors in parenthesis are clustered at the individual level.

Table A8. Effect of fast food restaurants on BMI, controlling for changes in residence ($D_{it}$)

|  | Home | |  | School | |
| --- | --- | --- | --- | --- | --- |
|  | (1) | (2) |  | (3) | (4) |
| Panel A. Fast food restaurants within |  |  |  |  |  |
| Equation 1, k = 400m | 0.108 | 0.0969 |  | 0.0242 | 0.0304 |
|  | (0.0758) | (0.0779) |  | (0.0432) | (0.0428) |
| Equation 1, k = 800m | 0.0544** | 0.0540** |  | 0.0696*** | 0.0750*** |
|  | (0.0257) | (0.0254) |  | (0.0222) | (0.0231) |
| Equation 1, k = 1600m | 0.0340*** | 0.0356*** |  | 0.0192* | 0.0227** |
|  | (0.0126) | (0.0126) |  | (0.00993) | (0.0106) |
| Panel B. Equation 2 |  |  |  |  |  |
| Fast food restaurants |  |  |  |  |  |
| within 400m | 0.110 | 0.101 |  | 0.0330 | 0.0412 |
|  | (0.0763) | (0.0777) |  | (0.0432) | (0.0428) |
| between 400m and 800m | 0.0400 | 0.0418 |  | 0.0776*** | 0.0825*** |
|  | (0.0304) | (0.0305) |  | (0.0237) | (0.0246) |
| between 800m and 1600m | 0.0266* | 0.0288** |  | 0.00387 | 0.00690 |
|  | (0.0141) | (0.0142) |  | (0.0113) | (0.0121) |
|  |  |  |  |  |  |
| Other food outlets | Yes | Yes |  | Yes | Yes |
| Individual controls | Yes | Yes |  | Yes | Yes |
| Area level controls | Yes | Yes |  | Yes | Yes |
| Individual FE | Yes | Yes |  | Yes | Yes |
| Year of survey FE | Yes | Yes |  | Yes | Yes |
| Change of residence control | No | Yes |  | No | Yes |
| Observations | 24,759 | 24,759 |  | 24,759 | 24,759 |
| Individuals | 8,253 | 8,253 |  | 8,253 | 8,253 |
| Mean of dependent variable | 18.95 | 18.95 |  | 18.95 | 18.95 |

Notes: Columns 2 and 4 show estimates of equations (1) and (2) controlling for changes in residence ($D_{it}$). Columns 1 and 3 show benchmark estimates for comparison purposes (similar to columns 4 and 8 in Table 3). In Panel A, each cell reports a different regression, and rows show results for three different equations – one for each respective buffer in equation (1): $\beta^{400}$, $\beta^{800}$ , and $\beta^{1600}$. In Panel B, rows show estimates of equation (2): $\gamma_{1}$,$\gamma_{2}$, and $\gamma_{3}$. Other estimates are omitted due to space limitations but available upon request. ***, **, and * denote statistically significant at 1%, 5% and 10%. Robust standard errors in parenthesis are clustered at the individual level.

Table A9. Effect of fast food restaurants around 1600 metres on BMI, controlling mode transport from school to home.

|  | 1600 m | | 800 m | | 400 m | |
| --- | --- | --- | --- | --- | --- | --- |
|  | Home | School | Home | School | Home | School |
|  | (1) | (2) | (3) | (4) | (5) | (6) |
| Fast food restaurants within 1600m | 0.0353*** | 0.0226** | 0.0564** | 0.0764*** | 0.0883 | 0.0406 |
|  | (0.0127) | (0.0105) | (0.0253) | (0.0226) | (0.0775) | (0.0426) |
| Mode transport from school to home |  |  |  |  |  |  |
| Public transport | 0.0397 | 0.0435 | 0.0378 | 0.0479 | 0.0390 | 0.0430 |
|  | (0.108) | (0.109) | (0.109) | (0.108) | (0.108) | (0.109) |
| School or local authority bus, minibus | 0.125 | 0.118 | 0.122 | 0.122 | 0.119 | 0.119 |
|  | (0.0791) | (0.0793) | (0.0792) | (0.0792) | (0.0793) | (0.0793) |
| Car or other vehicle | 0.198*** | 0.202*** | 0.199*** | 0.206*** | 0.199*** | 0.202*** |
|  | (0.0532) | (0.0531) | (0.0533) | (0.0533) | (0.0532) | (0.0532) |
| Bicycle | 0.124 | 0.106 | 0.115 | 0.114 | 0.115 | 0.108 |
|  | (0.125) | (0.128) | (0.128) | (0.129) | (0.128) | (0.128) |
| Other | -0.290* | -0.291* | -0.298* | -0.291* | -0.299** | -0.288* |
|  | (0.153) | (0.151) | (0.152) | (0.152) | (0.153) | (0.153) |
|  |  |  |  |  |  |  |
| Other Food Outlets | Yes | Yes | Yes | Yes | Yes | Yes |
| Individual controls | Yes | Yes | Yes | Yes | Yes | Yes |
| Area level economic controls | Yes | Yes | Yes | Yes | Yes | Yes |
| Individual FE | Yes | Yes | Yes | Yes | Yes | Yes |
| Year of survey FE | Yes | Yes | Yes | Yes | Yes | Yes |
| Observations | 24,615 | 24,615 | 24,615 | 24,615 | 24,615 | 24,615 |
| Individuals | 8,249 | 8,249 | 8,249 | 8,249 | 8,249 | 8,249 |
| Mean of the dependent variable | 18.94 | 18.94 | 18.94 | 18.94 | 18.94 | 18.94 |

Notes: This table show OLS estimates for our preferred individual fixed-effect specification including additional time-varying controls. Outcome is BMI and other estimates are omitted due to space restrictions but available upon request. ***, **, and * denote statistically significant at 1%, 5% and 10%. Robust standard errors in parenthesis are clustered at the individual level.

Table A10. Robustness analysis: Effect of a modified fast food restaurant classification on BMI, equation (1)

|  | Home | | School | |
| --- | --- | --- | --- | --- |
|  | (1) | (2) | (3) | (4) |
| Fast food restaurants (base classification) |  |  |  |  |
| Equation 1, k = 400m | 0.0980 |  | 0.0296 |  |
|  | (0.0775) |  | (0.0426) |  |
| Equation 1, k = 800m | 0.0554** |  | 0.0742*** |  |
|  | (0.0255) |  | (0.0229) |  |
| Equation 1, k = 1600m | 0.0355*** |  | 0.0225** |  |
|  | (0.0126) |  | (0.0106) |  |
| Fast food restaurants (modified classification) |  |  |  |  |
| Equation 1, k = 400m |  | 0.0861 |  | 0.0336 |
|  |  | (0.0758) |  | (0.0435) |
| Equation 1, k = 800m |  | 0.0509** |  | 0.0698*** |
|  |  | (0.0253) |  | (0.0221) |
| Equation 1, k = 1600m |  | 0.0369*** |  | 0.0214** |
|  |  | (0.0124) |  | (0.0106) |
|  |  |  |  |  |
| Other food outlets | Yes | Yes | Yes | Yes |
| Individual controls | Yes | Yes | Yes | Yes |
| Area level controls | Yes | Yes | Yes | Yes |
| Individual FE | Yes | Yes | Yes | Yes |
| Year of survey FE | Yes | Yes | Yes | Yes |
| Observations | 24,759 | 24,759 | 24,759 | 24,759 |
| Number of individuals | 8,253 | 8,253 | 8,253 | 8,253 |
| Mean of dependent variable | 18.95 | 18.95 | 18.95 | 18.95 |

Notes: ***, **, and * denote statistically significant at 1%, 5% and 10%. Robust standard errors in parenthesis are clustered at the individual level.

Table A11. Robustness analysis: Effect of a modified fast food restaurant classification on BMI, equation (2)

|  | Home | | School | |
| --- | --- | --- | --- | --- |
|  | (1) | (2) | (3) | (4) |
| Fast food restaurant (base classification) |  |  |  |  |
| within 400m | 0.102 |  | 0.0404 |  |
|  | (0.0773) |  | (0.0426) |  |
| between 400m and 800m | 0.0432 |  | 0.0818*** |  |
|  | (0.0306) |  | (0.0244) |  |
| between 800m and 1600m | 0.0283** |  | 0.00683 |  |
|  | (0.0142) |  | (0.0120) |  |
| Fast food restaurant (modified classification) |  |  |  |  |
| within 400m |  | 0.0887 |  | 0.0428 |
|  |  | (0.0744) |  | (0.0435) |
| between 400m and 800m |  | 0.0410 |  | 0.0757*** |
|  |  | (0.0314) |  | (0.0232) |
| between 800m and 1600m |  | 0.0315** |  | 0.00656 |
|  |  | (0.0140) |  | (0.0122) |
|  |  |  |  |  |
| Other food outlets | Yes | Yes | Yes | Yes |
| Individual controls | Yes | Yes | Yes | Yes |
| Area level controls | Yes | Yes | Yes | Yes |
| Individual FE | Yes | Yes | Yes | Yes |
| Year of survey FE | Yes | Yes | Yes | Yes |
| Observations | 24,759 | 24,759 | 24,759 | 24,759 |
| Number of individuals | 8,253 | 8,253 | 8,253 | 8,253 |
| Mean of dependent variable | 18.95 | 18.95 | 18.95 | 18.95 |

Notes: ***, **, and * denote statistically significant at 1%, 5% and 10%. Robust standard errors in parenthesis are clustered at the individual level.

Figure A1. Placebo estimates for child’s BMI, equation (1)


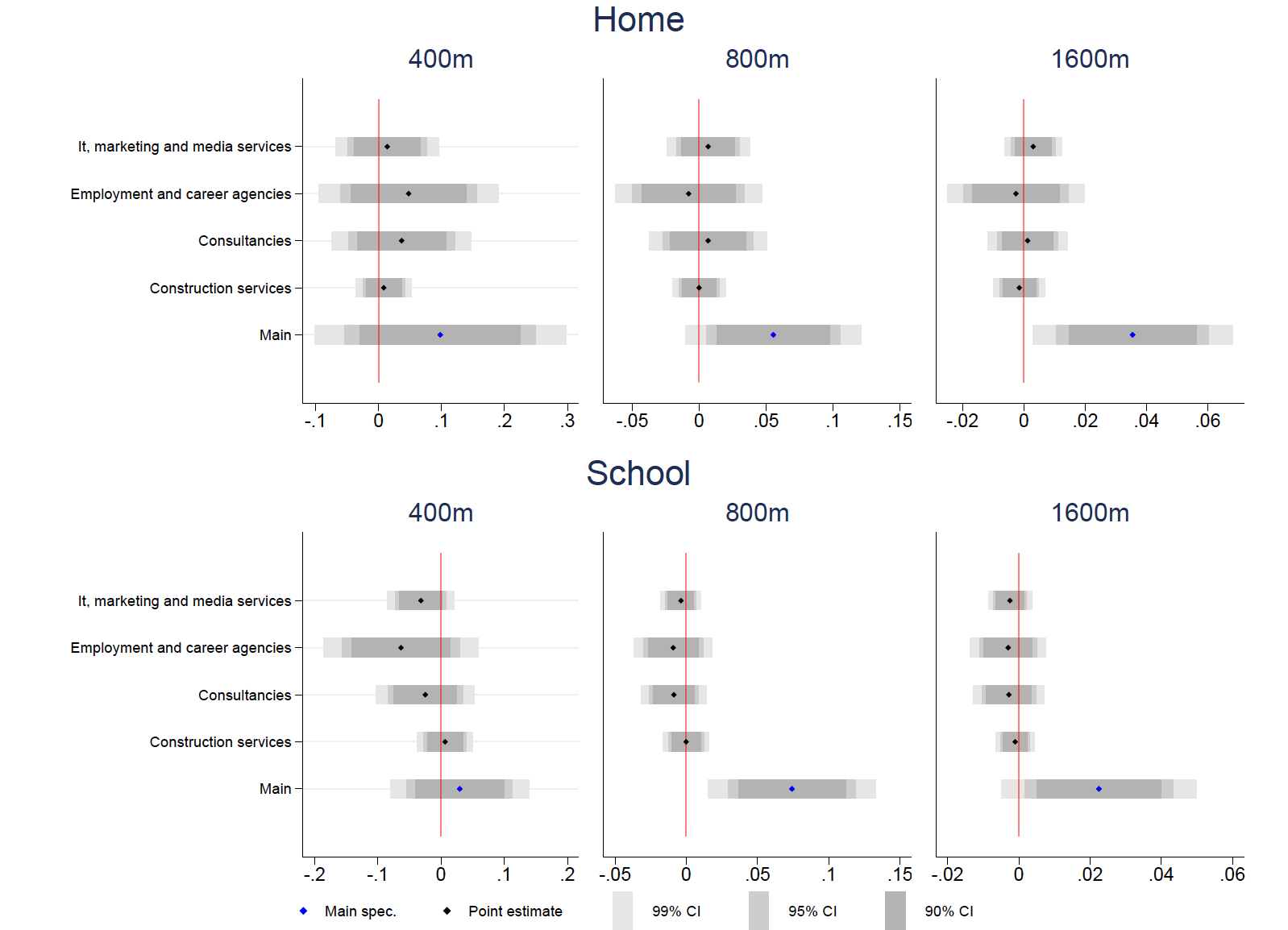


Notes: Each point estimate denotes a different regression using individuals’ BMI as depended variable. This figure shows estimates for equation (1) using our preferred individual fixed effect specification. Our preferred specification is labelled as ‘Main’. Other estimates shown in this figure replace the number of fast food restaurants by the numbers of other placebo PoI facilities within 400, 800, and 1600 metres from individual’s residence and school. We plot estimates using placebo exposure to facilities in the following PoI categories: ‘It, marketing and media services’, ‘Employment and career agencies’, ‘Consultancies’, and ‘Construction services.

Figure A2. Placebo estimates for child’s Body Fat percentage, equation (1)


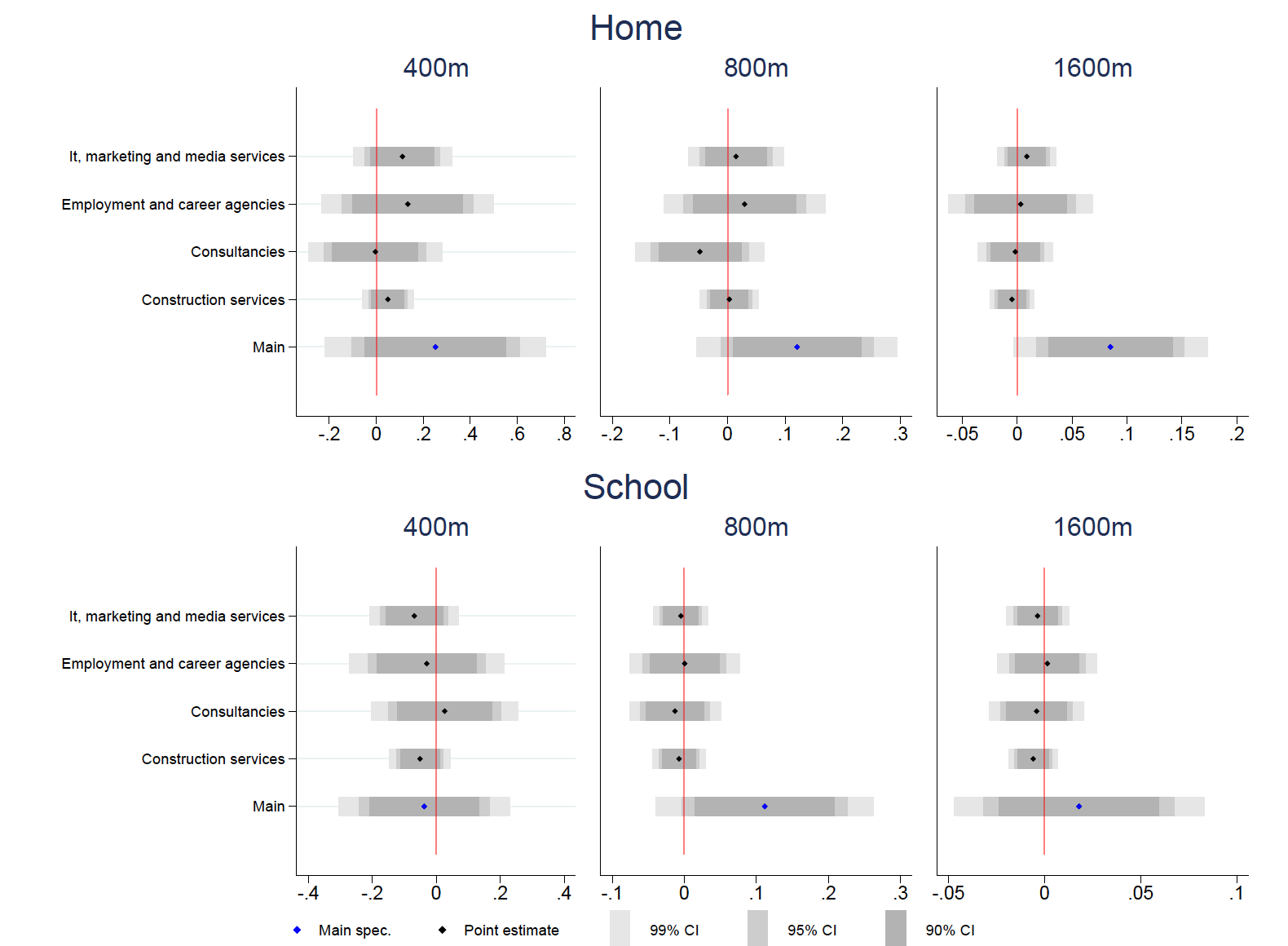


Notes: Each point estimate denotes a different regression using individuals’ Body Fat percentage as depended variable. This figure shows estimates for equation (1) using our preferred individual fixed effect specification. Our preferred specification is labelled as ‘Main’. Other estimates shown in this figure replace the number of fast food restaurants by the numbers of other placebo PoI facilities within 400, 800, and 1600 metres from individual’s residence and school. We plot estimates using placebo exposure to facilities in the following PoI categories: ‘It, marketing and media services’, ‘Employment and career agencies’, ‘Consultancies’, and ‘Construction services.

Figure A3. Placebo estimates for child’s BMI, equation (2)


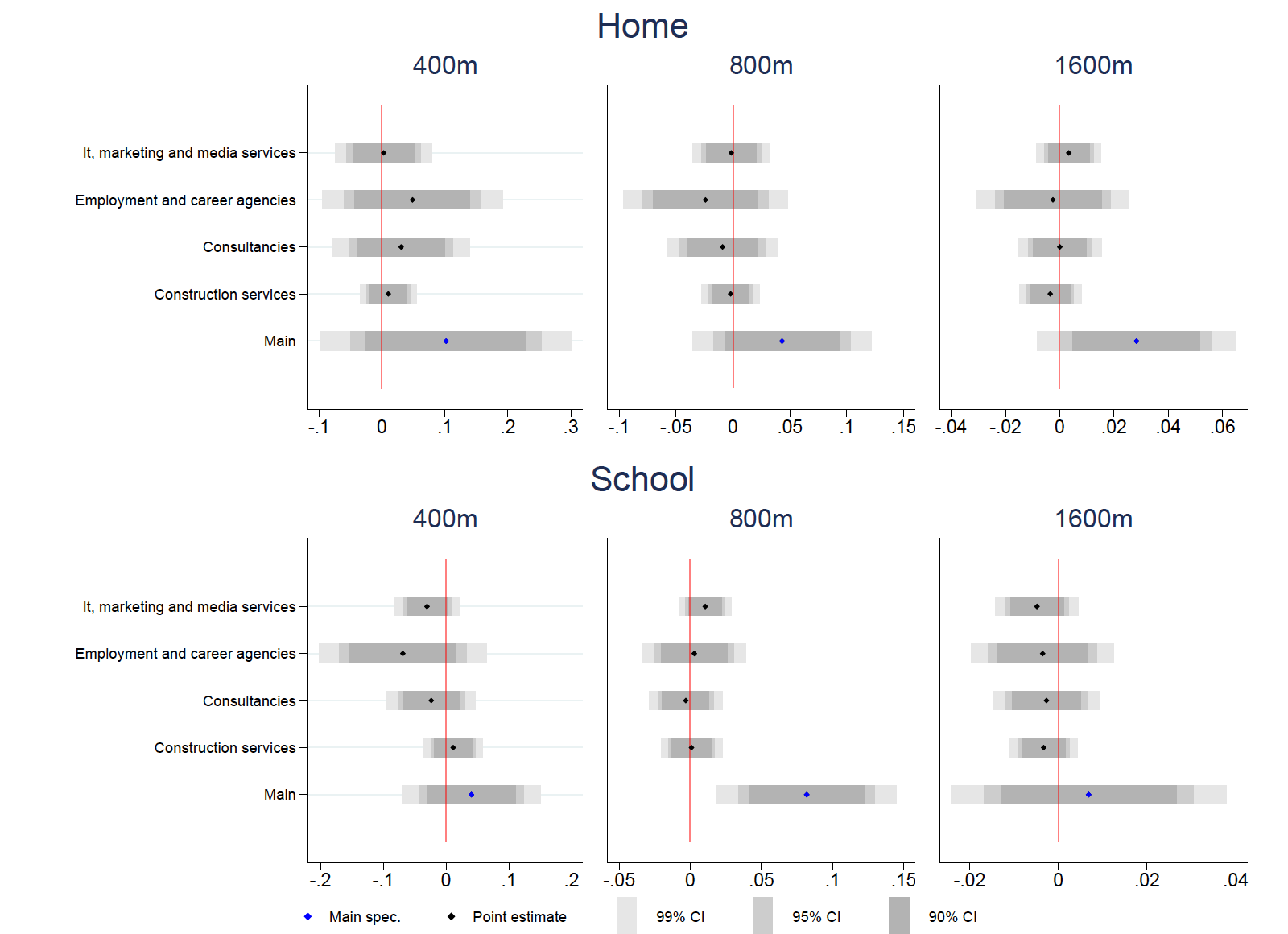


Notes: Each point estimate denotes a different regression using individuals’ BMI as depended variable. This figure shows estimates for equation (2) using our preferred individual fixed effect specification. Our preferred specification is labelled as ‘Main’. Other estimates shown in this figure, replace the number of fast food restaurants by the numbers of other placebo PoI facilities within 400 metres, between 400 and 800 metres, and between 800 and 1600 metres from individuals’ residences and schools. We plot estimates using placebo exposure to facilities in the following PoI categories: ‘It, marketing and media services’, ‘Employment and career agencies’, ‘Consultancies’, and ‘Construction services.

Figure A4. Placebo estimates for child’s Body Fat percentage, equation 2


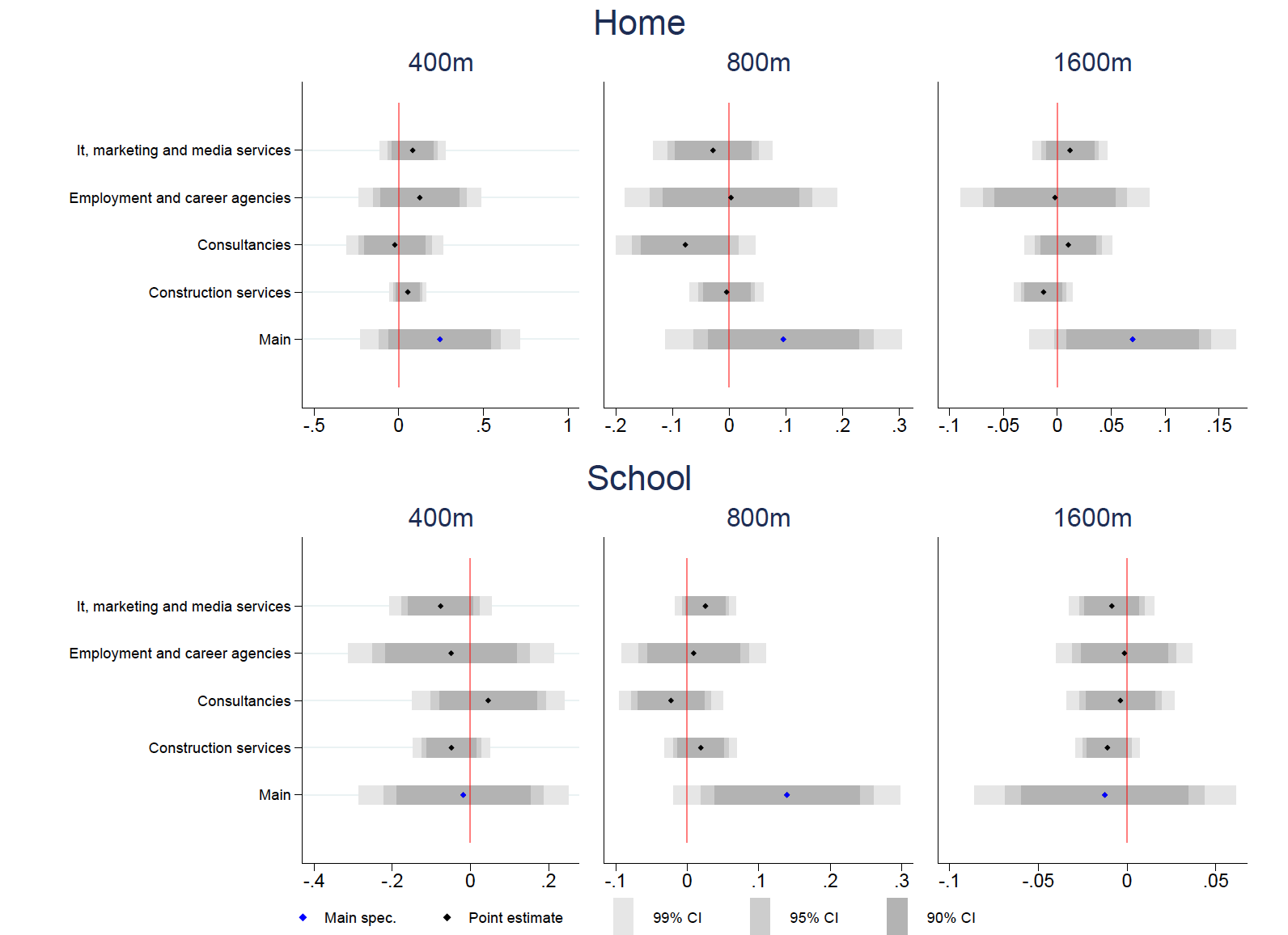


Notes: Each point estimate denotes a different regression using individuals’ Body Fat percentage as depended variable. This figure shows estimates for equation (2) using our preferred individual fixed effect specification. Our preferred specification is labelled as ‘Main’. Other estimates shown in this figure, replace the number of fast food restaurants by the numbers of other placebo PoI facilities within 400 metres, between 400 and 800 metres, and between 800 and 1600 metres from individuals’ residences and schools. We plot estimates using placebo exposure to facilities in the following PoI categories: ‘It, marketing and media services’, ‘Employment and career agencies’, ‘Consultancies’, and ‘Construction services.

Table A12. Association of Emotional Dysregulation at age 7 and outcomes at age 14 and 11

|  | Eat fast food one or more days per week (age 14) | Ever smoke (age 14) | Ever tried alcohol (age 14) | Individual’s Patience (age 14) | Risk taking (age 11) |
| --- | --- | --- | --- | --- | --- |
|  | (1) | (2) | (3) | (4) | (5) |
| Panel A. |  |  |  |  |  |
| High emotional dysregulation | 4.478*** | 5.004*** | 4.132*** | -0.554*** | 0.0145*** |
|  | (1.194) | (1.411) | (1.402) | (0.0601) | (0.00434) |
| Panel B. |  |  |  |  |  |
| Emotional dysregulation | 3.935*** | 6.831*** | 5.264*** | -0.726*** | 0.0171*** |
|  | (1.393) | (1.519) | (1.611) | (0.0754) | (0.00476) |
|  |  |  |  |  |  |
| Observations | 8,029 | 7,643 | 7,710 | 8,001 | 7,719 |
| Individual controls at age 7 | Yes | Yes | Yes | Yes | Yes |
| Area level economic controls at age 7 | Yes | Yes | Yes | Yes | Yes |
| Mean of the dependent variable | 26.85 | 52.91 | 50.80 | 5.708 | 0.527 |

Notes: This table show OLS estimates. Each cell reports a different regression. Individual and area level controls at age 7 are the same we include in our main specification. The outcome in column 1 is a dummy variable that measures if the individual eats fast food one or more days per week. In columns 2 and 3, outcomes variables are dummy variables indicating if the individual has ever smoked and drank alcohol, respectively. Individual’s Patience is a continuous variables created with the question ‘How patient is the respondent?’. It is a score ranging from 0 to 10, where 10 indicates that the highest level of patience. Risk taking at age 11 is measured using the risk taking score of the CANTAB Cambridge Gambling Task, where higher values indicate of greater risk taking ([Atkinson, 2015](#_ENREF_1)). Dependent are shown in Panel A and B. High emotional dysregulation is a dummy variable equals 1 if the emotional dysregulation sub-scale of the Child Social Behaviours Questionnaire at age 7 is above the sample median and 0 otherwise. The Emotional dysregulation score is the score of the dysregulation sub-scale of the Child Social Behaviours Questionnaire at age 7.

Table A13. Effect of fast food restaurants on individual’s diet and take-up of school meals.

|  | (1) | (2) | (3) | (4) | (5) |
| --- | --- | --- | --- | --- | --- |
|  | Do not eat fruits | Drink sweetened drinks | Skip breakfast at least one time per week | Unhealthy diet score | Meal is provided by school |
|  |  |  |  |  |  |
| Panel A. Fast food restaurants within 1600m from homes | -0.151 | 0.393 | 0.190 | 0.00447 | -0.213 |
|  | (0.134) | (0.322) | (0.246) | (0.00426) | (0.268) |
|  |  |  |  |  |  |
| Panel B. Fast food restaurants within 1600m from schools | -0.0397 | 0.076 | -0.053 | 0.00002 | -0.197 |
|  | (0.0979) | (0.234) | (0.173) | (0.00291) | (0.213) |
|  |  |  |  |  |  |
| Other Food Outlets | Yes | Yes | Yes | Yes | Yes |
| Individual controls | Yes | Yes | Yes | Yes | Yes |
| Area level economic controls | Yes | Yes | Yes | Yes | Yes |
| Individual FE | Yes | Yes | Yes | Yes | Yes |
| Observations | 24,680 | 24,566 | 24,689 | 24,698 | 24,105 |
| Number of individuals | 8,253 | 8,253 | 8,253 | 8,253 | 8,249 |
| Mean of the dependent variable | 6.22% | 47.74% | 20.66% | 0.744 | 54.56% |

Notes: This table show OLS results for equation (1) but replacing BMI by diet variables. Coefficients in columns 1, 2, 3, and 5 indicate percentage points changes. Outcome variables are defined as follow. ‘Do not eat fruits’ is a dummy variable equals to 100 if individuals do not report eating fruit and 0 otherwise. In the MCS sweeps 4 and 5, ages 7 and 11, we use the question ‘On a typical day, how many portions of fresh, frozen, tinned, or dried fruit does the individual eat?’, and we define *Consumption of fruits* as those who reported one or more portions; however, in the MCS sweep 6, age 14, we use the question ‘How often do you eat at least 2 portions of fruit per day? A portion of fruit could be a whole piece of fruit, like an apple or banana or 80g of fruit (like in a fruit salad) but does not include fruit juices’, and we define *Consumption of fruits* as those who reported ‘Some days, but not all days’ or ‘Every day’. Given the change in the question between surveys we are only able to proxy whether the children eat or not fruits, but not the frequency of fruit consumption. ‘Drink sweetened drinks’ is a dummy variable equals to 100 if respondent drink sweetened drinks and 0 otherwise. In the MCS sweep 4, age 7, parents were asked ‘When the individuals drinks between meals, what does he/she drinks?’. For age 7, we defined *Consumption* of *sweetened drinks* as a binary variable indicating whether individual reported drinking ‘Artificially sweetened drinks (diet cola, sugar-free squash)’. However, in the MCS sweep 5 and 6, ages 11 and 14, individuals were asked ‘How often, if at all, do you drink sugary drinks like regular cola or squash?’. For ages 11 and 14, we defined *Consumption* of *sweetened drinks* as a binary variable indicating whether individual reported one or more days a week. Given the change in the question between surveys we are only able to proxy whether the children drink or not *sweetened drinks*, but not the frequency of consumption. We use the question ‘How often do you eat breakfast over a week?’ to define those children who skip breakfast at least one day per week. *Unhealthy* *diet* score is defined as the sum of the three previous dummy variables, with higher scores indicating poorer quality diets. ‘Meal is provided by school’ is a dummy variable equals to 100 if the individual’s meal was provided by the school and 0 otherwise. Each cell reports a different regression. Panel A show estimates around homes and Panel B around school. Other estimates in equation (1) are omitted due to space limitations but available upon request. ***, **, and * denote statistically significant at 1%, 5% and 10%. Robust standard errors in parenthesis are clustered at the individual level.

Table A14. Effect of fast food restaurants on individuals’ physical activity.

|  | (1) | (2) | (3) | (4) |
| --- | --- | --- | --- | --- |
|  | Physical Activity (continuous) | 1 day or more | 3 days or more | 5 days or more |
|  |  |  |  |  |
| Panel A. Fast food restaurants within 1600m from homes | 0.00408 | 0.161 | -0.138 | 0.177 |
|  | (0.00995) | (0.288) | (0.241) | (0.202) |
|  |  |  |  |  |
| Panel B. Fast food restaurants within 1600m from schools | 0.00954 | 0.208 | 0.101 | 0.176 |
|  | (0.00766) | (0.192) | (0.202) | (0.151) |
|  |  |  |  |  |
| Other Food Outlets | Yes | Yes | Yes | Yes |
| Individual controls | Yes | Yes | Yes | Yes |
| Area level economic controls | Yes | Yes | Yes | Yes |
| Individual FE | Yes | Yes | Yes | Yes |
| Observations | 24,703 | 24,703 | 24,703 | 24,703 |
| Number of individuals | 8,253 | 8,253 | 8,253 | 8,253 |
| Mean of the dependent variable | 2.467 | 80.28 | 42.22 | 16.74 |

Notes: This table show OLS results for equation (1) but replacing BMI by physical activity variables. The outcome variables in Columns 2, 3, and 4 are three binary variables that indicate whether the individual exercised 1 or more, 3 or more, and 5 or more days per week. The outcome variable in Column 1 is a continuous variable that imputes the mid-point of the intervals associated to each category (i.e., zero days, 1.5 days, 3.5 days, and 6 days). Each cell reports a different regression. Panel A show estimates around homes and Panel B around school. Other estimates in equation (1) are omitted due to space limitations but available upon request. ***, **, and * denote statistically significant at 1%, 5% and 10%. Robust standard errors in parenthesis are clustered at the individual level

Figure A5. Effect of fast food restaurants around 1600 metres from MCS residences compared with de Chaisemartin & D’Haultfoeuille (2020) estimator.


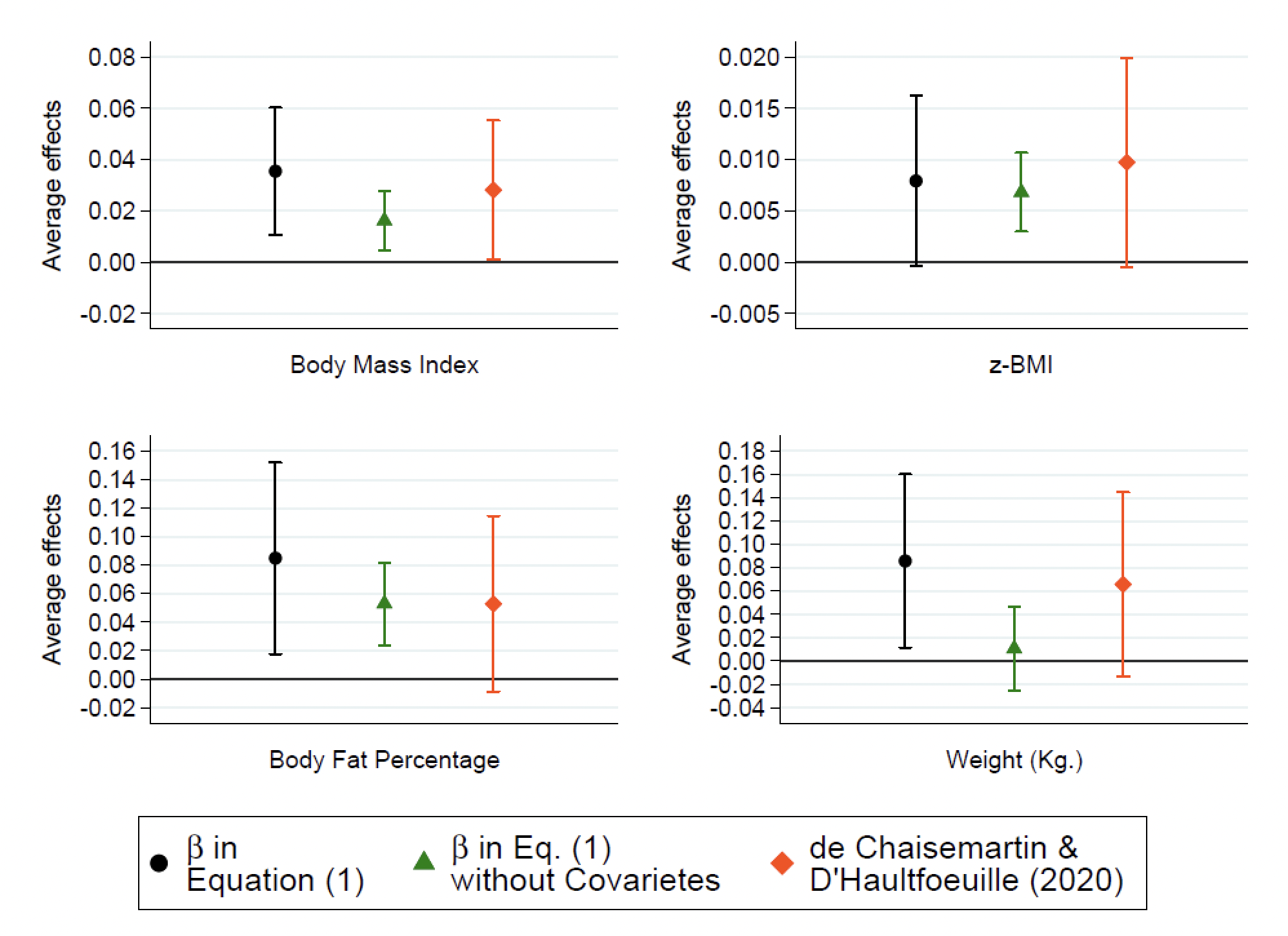


Notes: This Figure overlay three estimators: The FE estimator in Equation (1) that controls for individual and area level characteristics (in black with circle markers), the FE estimator in Equation (1) without covariates (in green with triangle markers), and de Chaisemartin and d’Haultfoeuille (2020) (in orange with diamonds markers). In order to estimate standard error, de Chaisemartin and d’Haultfoeuille estimator does not include controls as our treatment is non-binary and for some values the number of controls exceed the sample size. The bars represent 95 percent confidence intervals. Confidence intervals for de Chaisemartin and d’Haultfoeuille estimator were computed with 200 bootstrap replications. Standard errors are clustered at the individual level.

Figure A6. Effect of fast food restaurants around 1600 metres from MCS schools compared with de Chaisemartin & D’Haultfoeuille (2020) estimator.


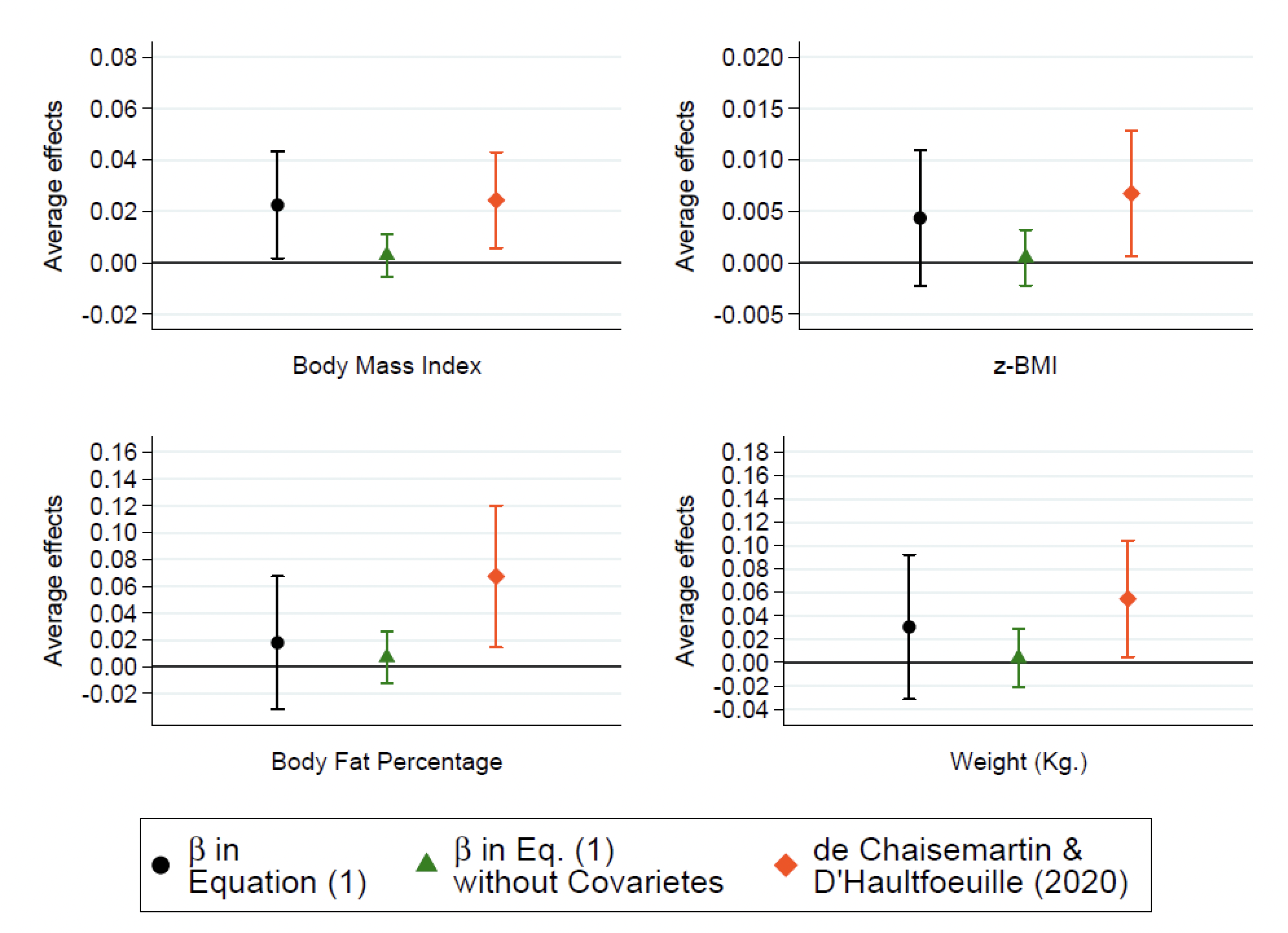


Notes: This Figure overlay three estimators: The FE estimator in Equation (1) that controls for individual and area level characteristics (in black with circle markers), the FE estimator in Equation (1) without covariates (in green with triangle markers), and de Chaisemartin and d’Haultfoeuille (2020) (in orange with diamonds markers). In order to estimate standard error, de Chaisemartin and d’Haultfoeuille estimator does not include controls as our treatment is non-binary and for some values the number of controls exceed the sample size. The bars represent 95 percent confidence intervals. Confidence intervals for de Chaisemartin and d’Haultfoeuille estimator were computed with 200 bootstrap replications. Standard errors are clustered at the individual level.

**Appendix B**

**Definitions of covariates, fast food restaurants and other food outlets.**

**Fast food restaurants**

The main fast food restaurant variable used in this paper is defined as the number of the following fast food chains in the Points of Interest data.

- McDonalds: The outlet’s name in the PoI data includes the string “McDonald’s Restau*” OR “McDonalds” AND belongs to the PoI group “Accommodation, eating and drinking” and category “Eating and drinking”.
- KFC: The outlet’s name in the PoI data includes the string “KFC” OR “K F C*” OR “KFC*” AND belongs to the PoI group “Accommodation, eating and drinking” and category “Eating and drinking”.
- Burger King: The outlet’s name in the PoI data includes the string “Burger King*” AND belongs to the PoI group “Accommodation, eating and drinking” and category “Eating and drinking”.
- Wimpy: The outlet’s name in the PoI data includes the string “Wimpy*” AND belongs to the PoI group “Accommodation, eating and drinking” and category “Eating and drinking”.
- Subway: The outlet’s name variable in the PoI data includes the string “Subway*” AND belongs to the PoI group “Accommodation, eating and drinking” and category “Eating and drinking”.
- Pizza Hut: The outlet’s name in the PoI data includes the string “*Pizza Hut*” AND belongs to the PoI group “Accommodation, eating and drinking” and category “Eating and drinking”.
- Dominos’ Pizza: The outlet’s name in the PoI data includes the string “Domino’s Pizza*” AND belongs to the PoI group “Accommodation, eating and drinking” and category “Eating and drinking”.
- Kebab and Chicken: The outlet’s name in the PoI data includes the string (“*Chicken*” OR “*Keba*”) AND belongs to the PoI group “Accommodation, eating and drinking” and category “Eating and drinking”.
- Fish and chip shops: All outlets belonging to the class 1020020 “Fish and chip shop”, of the PoI group “Accommodation, eating and drinking” and category “Eating and drinking”.

The decision to include fish and chips, and kebab and chicken outlets, is driven by both context and content: as salient fast food types across the UK, they provide highly calorific and processed meals ([Jaworowska et al., 2014](#_ENREF_8)). Fish and chips shops and chicken outlets have been classified as unhealthy outlets in previous literature ([Cetateanu & Jones, 2014](#_ENREF_4); [Wilkins, Morris, Radley, & Griffiths, 2019](#_ENREF_11)). In 2006, the UK’s Food Standards Agency (FSA) found that 18.5% of doner kebabs constitute a “significant” threat to public health ([FSA, 2006](#_ENREF_6)), while a 2009 study found that 97%, 98% and 96% of kebabs would be unhealthy for their fat, saturates fat, and salt content, respectively ([LACORS, 2009](#_ENREF_9)). The authors found that “the average kebab provides men (women) with 66% (89%) of their Guideline Daily Amount (GDA) of fat, 98% (148%) of their GDA for saturated fats and 98% (98%) of the GDA for salt”.

**Other food outlets**

Using the Points of Interest classification scheme v3.1. The ‘Other food outlets’ variable (Table A1 and A2 in Online Appendix) is defined as the number of outlets in the following classes:

- From the PoI group ‘Accommodation, eating and drinking’ and category ‘Eating and drinking’
  - 1020043: Restaurants
  - 1020018: Takeaway outlets and 1020019: Food delivery services. These categories were grouped in a category named ‘Other takeaway outlets’
- From the PoI group ‘Retail’, and category ‘Food, drink and multi item retail’
  - 9470661: Bakeries
  - 9470662: Butchers
  - 9470663: Confectioners
  - 9470665: Delicatessens
  - 9470666: Fishmongers
  - 9470668: Green and new age goods
  - 9470669: Grocers; farm shops and pick your own
  - 9470672: Organic; health; gourmet and kosher foods
  - 9470699: Convenience stores and independent supermarkets
  - 9470819: Supermarket chains

**Controls used in equations (1) and (2)**

**Individual level control**

1. Maternal highest educational level: We use variable National Vocational Qualification (NVQ) variable created by CLS and available in the public data. Looks at academic and vocational qualifications gained by the MAIN respondent since last interview and compares them with the derived NVQ highest level from previous sweeps to ascertain the overall highest level attained across all sweeps. More details can be found in the documents “MCS: Guide to Derived Variables for waves 3, 4, 5 and 6”.
2. Number of Parents/Careers in household: We use variable HTYS created by CLS and available in the public data. Is a collapsed version of HTYP into a 1 or 2 parent family:
3. *Two parents/carers*
4. *One parent/carer*

More details can be found in the documents “MCS: Guide to Derived Variables for waves 3, 4, 5 and 6”.

1. OECD equivalised weekly family income: We use variable OEDE, created by CLS and available in the public data. It divides total net income by number of household members according to their weight on the OECD equivalised income scale (equivalised household size) to give net disposable income. More details can be found in the documents “MCS: Guide to Derived Variables for waves 3, 4, 5 and 6”.
2. Number of people in household (not including individual): We use variable NUMH created by CLS and available in the public data. It uses the variable PRES from the household grid to count the number of people present in the household (but does not include CMs) More details can be found in the documents “MCS: Guide to Derived Variables for waves 3, 4, 5 and 6”.
3. Numbers of rooms in the household: We use the question ROMA.

*ROMA: How many rooms do you and your family have here excluding bathrooms, toilets, halls and garages?*

**Area level controls**

1. Unemployment rate: We linked the unemployment rate of local authority districts using MCS respondent’s postcode of residence at each interview.

Source: <https://www.nomisweb.co.uk/query/construct/summary.asp?mode=construct&version=0&dataset=127>

1. Population estimates per 100,000 people: We linked annual population estimates at local authority districts level using MCS respondent’s postcode of residence at each interview.

Source: https://www.nomisweb.co.uk/query/construct/summary.asp?mode=construct&version=0&dataset=2002

Table B1. PoI categories used in falsification tests

| **Group description** | **Category description** | **Class description** |
| --- | --- | --- |
| Commercial services | Construction services | Metalworkers including blacksmiths |
|  |  | Building contractors |
|  |  | Construction completion services |
|  |  | Construction plant |
|  |  | Cutting, drilling and welding services |
|  |  | Demolition services |
|  |  | Diving services |
|  |  | Electrical contractors |
|  |  | Gardening, landscaping and tree surgery services |
|  |  | Glaziers |
|  |  | Painting and decorating services |
|  |  | Plasterers |
|  |  | Plumbing and heating services |
|  |  | Pool and court construction |
|  |  | Restoration and preservation services |
|  |  | Road construction services |
|  |  | Roofing and chimney services |
|  |  | Fencing and drystone walling services |
|  |  | Building and component suppliers |
| Commercial services | Consultancies | Architectural and building related consultants |
|  |  | Business related consultants |
|  |  | Computer consultants |
|  |  | Construction service consultants |
|  |  | Feng shui consultants, furnishers and shop fitters |
|  |  | Food consultants |
|  |  | Image consultants |
|  |  | Interpretation and translation consultants |
|  |  | Security consultants |
|  |  | Telecommunications consultants |
|  |  | Traffic management and transport related consultants |

Table B1. PoI categories used in definition of Falsification tests (cont.)

| **Group description** | **Category description** | **Class description** |
| --- | --- | --- |
| Commercial services | Employment and career agencies | Careers offices and armed forces recruitment |
|  |  | Domestic staff and home help |
|  |  | Driver agencies |
|  |  | Employment agencies |
|  |  | Modelling and theatrical agencies |
|  |  | Nursing agencies |
| Commercial services | It, advertising, marketing and media services | Advertising services |
|  |  | Artists, illustrators and calligraphers |
|  |  | Computer security |
|  |  | Computer systems services |
|  |  | Concert/exhibition organisers and services |
|  |  | Database services |
|  |  | Desktop publishing services |
|  |  | Electronic and internet publishers |
|  |  | Film and video services |
|  |  | General computer services |
|  |  | Internet services |
|  |  | Literary services |
|  |  | Mailing and other information services |
|  |  | Marketing services |
|  |  | Plate makers, print finishers and type setters |
|  |  | Press and journalism services |
|  |  | Printing and photocopying services |
|  |  | Recording studios and record companies |
|  |  | Telephone, telex and fax services |
|  |  | Television and radio services |

**References**

Atkinson, M. (2015). *Interpreting the CANTAB cognitive measures*. CLS Data Note.

Borusyak, K., Jaravel, X., & Spiess, J. (2021). Revisiting Event Study Designs: Robust and Efficient Estimation. doi:10.48550/arxiv.2108.12419

Callaway, B., & Sant’Anna, P. H. C. (2021). Difference-in-Differences with multiple time periods. *Journal of Econometrics, 225*(2), 200-230. doi:<https://doi.org/10.1016/j.jeconom.2020.12.001>

Cetateanu, A., & Jones, A. (2014). Understanding the relationship between food environments, deprivation and childhood overweight and obesity: Evidence from a cross sectional England-wide study. *Health and Place, 27*, 68-76. doi:10.1016/j.healthplace.2014.01.007

de Chaisemartin, C., & D’Haultfœuille, X. (2020). Two-Way Fixed Effects Estimators with Heterogeneous Treatment Effects. *The American economic review, 110*(9), 2964-2996. doi:10.1257/aer.20181169

FSA. (2006). *Salt reduction targets*. Retrieved from Food Standards Agency: <http://www.food.gov.uk/multimedia/pdfs/salttargetsapril06.pdf>

Goodman-Bacon, A. (2021). Difference-in-differences with variation in treatment timing. *Journal of Econometrics, 225*(2), 254-277. doi:10.1016/j.jeconom.2021.03.014

Jaworowska, A., Blackham, T. M., Long, R., Taylor, C., Ashton, M., Stevenson, L., & Davies, I. (2014). Nutritional composition of takeaway food in the UK. *Nutrition & Food Science, 44*(5). doi:10.1108/NFS-08-2013-0093

LACORS. (2009). *The composition and labelling of Doner Kebabs*. Retrieved from <http://www.lanotte-dietista.it/blog/wp-content/uploads/2014/10/kebabcomposition-110318155214-phpapp01.pdf>

Sun, L., & Abraham, S. (2021). Estimating dynamic treatment effects in event studies with heterogeneous treatment effects. *Journal of Econometrics, 225*(2), 175-199. doi:10.1016/j.jeconom.2020.09.006

Wilkins, E., Morris, M., Radley, D., & Griffiths, C. (2019). Methods of measuring associations between the Retail Food Environment and weight status: Importance of classifications and metrics. *SSM Popul Health*. doi:10.1016/j.ssmph.2019.100404
